# Assessing large language model responses to pediatric depression FAQs: a cross-sectional study on readability, accuracy, and sentiment

**DOI:** 10.3389/fpsyt.2026.1782288

**Published:** 2026-04-29

**Authors:** RongQi Jiao, MingZhu Chen, Jing Zhang

**Affiliations:** Children's Hospital of Nanjing Medical University, Nanjing, China

**Keywords:** Adolescent depression, ChatGPT, Copilot GPT, DeepSeek, emotional tone analysis, large language models, readability assessment, sentiment analysis

## Abstract

**Background:**

Pediatric depression shows age-specific symptoms that hinder recognition and delay care, while parents and adolescents increasingly turn to online sources, including large language models, for mental health information and guidance. The quality of such information depends on readability, factual accuracy, completeness, and emotional tone. This study compared responses from 3 contemporary large language models (LLMs) to frequently asked questions about pediatric depression to assess their suitability as informational tools.

**Methods:**

A cross-sectional analytical study design was used. 15 standardized frequently asked questions covering definition, causes, clinical features, diagnosis, prevention, treatment, and prognosis of pediatric depression were submitted to ChatGPT-5, Microsoft Copilot GPT-5 in Smart Research mode, and DeepSeek 3.1V. Responses were collected verbatim. Readability was assessed using seven established indices. Accuracy and completeness were independently scored on a 0 to 6 scale using a predefined rubric. Sentiment was measured with sentiment scores. One-way analysis of variance (ANOVA) with Tukey *post hoc* statistical analysis was performed.

**Results:**

Readability was different among the various models. DeepSeek 3.1V achieved the highest Flesch Reading Ease Score of 54 to 55 and the lowest Flesch–Kincaid Grade Level of about 9.5 thus indicating easier comprehension. ChatGPT-5 showed intermediate readability with scores of 49 to 50 and grade level about 10.5. Copilot-5 had the lowest Reading Ease score of 43 to 44 and the highest grade level near 10.8. Accuracy on a 0 to 6 scale was highest for Copilot-5. ChatGPT-5 showed the greatest completeness, whereas other models had variable coverage in detailed clinical items.

**Conclusion:**

Large language models (LLMs) provide information on pediatric depression but show varying levels of readability, accuracy, and completeness. DeepSeek 3.1V provides greater linguistic accessibility, Microsoft Copilot GPT-5 shows stronger factual consistency, and ChatGPT-5 provides more comprehensive coverage. These artificial intelligence (AI) chatbot systems require human understanding before use in pediatric mental health education or guidance.

## 1 Introduction

Depression in children and adolescents is a major public health concern with various effects on emotional development, academic accomplishment, family functioning, and long-term mental health outcomes (1–3). The clinical expression during childhood and adolescence often differs from that of depression in adults. Irritability, behavioral disturbances, somatic complaints, and academic deterioration are the frequent dominant presentations that are the classic depressive symptoms (4, 5). These age-specific features lead to delayed recognition, since the symptoms are often attributed to the developmental changes or peer pressures. Depression during childhood and adolescence often remains untreated and is associated with recurrence in adulthood, along with increased risk of suicidal behavior, substance use, and long-term psychosocial impairment (1, 6). These outcomes highlight the need for accurate and accessible mental health information tailored to developmental stages. Parents and adolescents seek reliable information alongside clinical consultations, and online resources are frequently used to understand symptoms, diagnostic processes, and treatment options (5, 7). Digital platforms influence mental health education, particularly in settings where stigma, limited awareness, and restricted access to specialists reduce timely professional consultation. The quality of online information influences care-seeking decisions. Inaccurate or poorly structured content may increase anxiety, reinforce misconceptions, or delay appropriate intervention (8, 9). Thus, pediatric depression requires proper communication that preserves clarity, reassurance, and clinical accuracy while addressing the sensitive emotional concerns relevant to children and adolescents.

Large language models (LLMs) are artificial intelligence (AI) systems that are designed to generate human-readable text for tasks such as health information retrieval and patient education. Platforms like ChatGPT, Microsoft Copilot, and DeepSeek provide medical explanations through conversational responses to user queries (10, 11). Their accessibility has increased use in several medical fields. Previous studies have evaluated their performance in orthopedics, anesthesia, asthma, myopia prevention, and psychiatry, reporting differences in factual reliability, readability, and suitability for non-specialist readers (12–18). Studies on mental health have shown that LLMs can answer depression related questions and identify the depressive language patterns in text-based materials (19–22). There is variation in content depth, clinical accuracy, and emotional tone which is of interest when the information reaches these children and adolescents without professional supervision (23–25).

Readability defines the level of comprehension achieved by intended users of AI-generated health information. The responses produced by AI and LLM often require advanced reading proficiency, frequently exceeding the recommended levels for adolescents and caregivers with limited health knowledge (9, 13, 16). Complex sentence structures and vocabulary in AI and LLM outputs can reduce the understanding and limit their educational value. Thus, accuracy and completeness are essential indicators of quality for AI generated medical content. The performance levels in child and adolescent mental health shows adequate responses to general knowledge questions but demonstrate reduced quality in detailed clinical matters, clear diagnostic differentiation, and specific treatment protocol (20, 23, 24, 26). Incomplete discussion of risk factors, therapeutic options, or safety issues may lead to misinterpretation; therefore, structured comparative evaluation is necessary to study the differences in the performance among various questions (23, 27).

Emotional tone plays a significant role in mental health communication. Language that conveys reassurance and empathy may promote help-seeking and reduce emotional distress, whereas neutral or negatively framed responses from artificial intelligence systems may reduce engagement (19, 26). Pediatric depression presents a sensitive context, requiring careful evaluation of sentiment and tone. Accuracy and completeness are central indicators of quality in artificial intelligence generated medical content. Evidence in child and adolescent mental health shows adequate performance for general knowledge questions but lower performance in detailed clinical issues, diagnostic differentiation, and treatment guidance (20, 23, 24, 26). Incomplete discussion of risk factors, therapeutic options, or safety considerations may result in misunderstanding. Performance differences across question types highlight the importance of structured comparative assessment (23, 27). Most healthcare evaluations of large language models have focused on adult mental health, broad psychiatric knowledge, or specific tasks such as symptom detection (19, 20, 22). Some studies have incorporated readability, factual accuracy, completeness, and sentiment within a single framework for pediatric depression queries relevant to caregivers and adolescents (13, 16, 18). A structured evaluation of LLM responses to pediatric depression queries is necessary to determine their strengths and limitations and inform clinical, research, and policy decisions in pediatric mental health.

The present study was designed to systematically compare the responses generated by ChatGPT-5, Microsoft Copilot GPT-5, and DeepSeek 3.1V to standardize the frequently asked questions on pediatric depression. The primary objective was to evaluate the readability, accuracy, completeness, and sentiment of AI-generated responses using validated metrics. The study aims to clarify performance differences among contemporary models and guide the appropriate use of AI in pediatric mental health information delivery.

## 2 Methods

### 2.1 Study design

**Figure 1:**
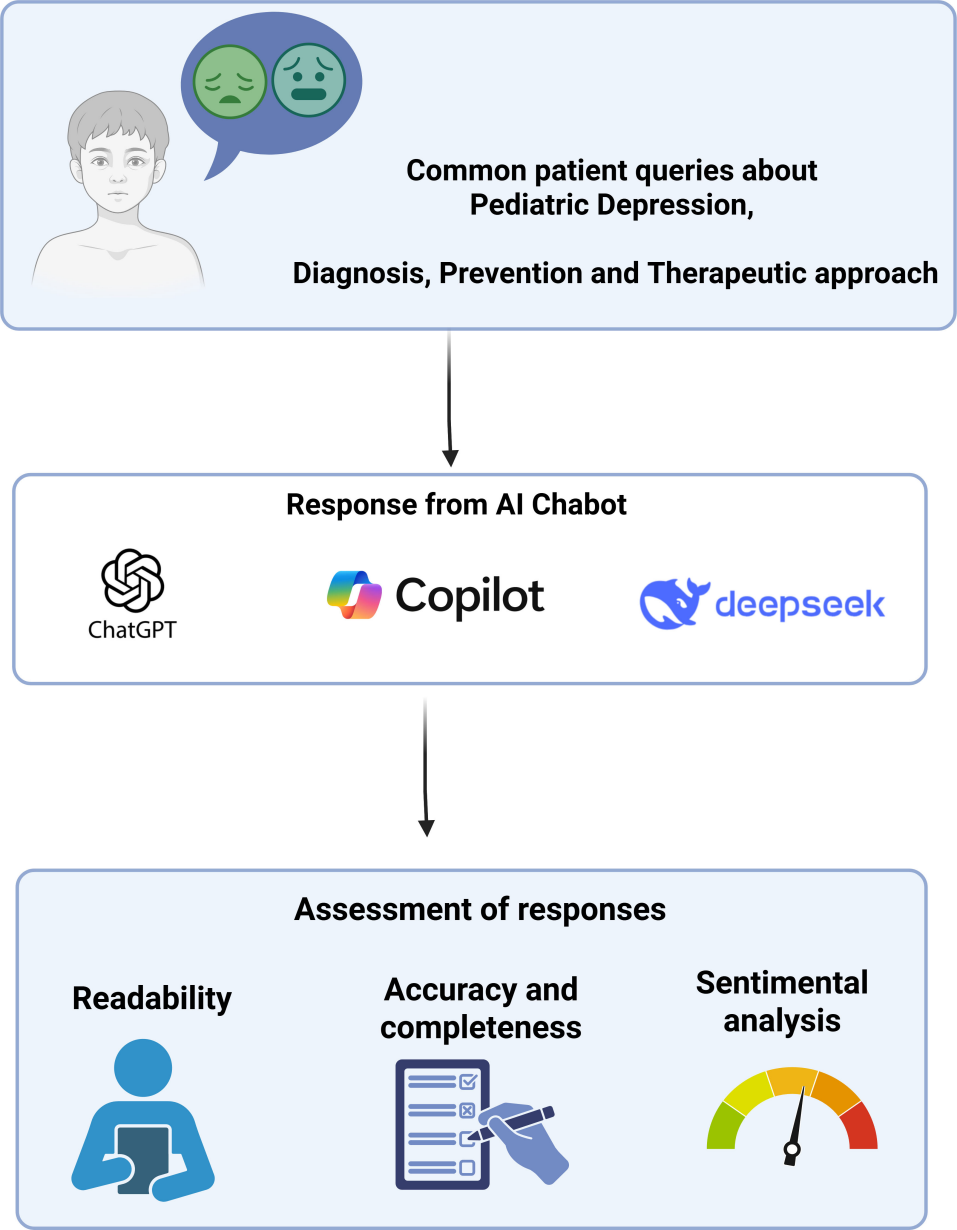
Flowchart showing the assessment of LLM responses to common pediatric depression queries, assessing readability, accuracy, and sentiment.

This cross-sectional analytical study evaluated the quality of responses generated by LLMs to frequently asked questions related to pediatric depression. The methodological workflow followed a predefined and standardized process and is summarized in **Figure 1**.

### 2.2 Selection of questions

15 standardized frequently asked questions were developed to represent the core clinical and educational domains of pediatric depression. These domains covered definition, types, risk factors, causes, clinical features, diagnostic approach, prevention strategies, treatment options, and prognosis. The questions were finalized before response generation and remained identical for all the three platforms to ensure consistency and comparability. The complete list of questions is provided in **Supplementary Table 1**. The 3 LLMs were selected for evaluation were ChatGPT-5, Microsoft Copilot GPT-5, and DeepSeek 3.1V. All models were accessed through their publicly available interfaces during the same study period to minimize temporal variability and ensure comparability of responses.

### 2.3 Response generation and data collection

These fifteen questions were independently submitted to all three models without modification or follow-up prompts. Responses were generated in a single iteration per question per model. All outputs were captured verbatim and entered into a structured Microsoft Excel database. The database included identifiers for the model, question number, and full response text. No post processing, editing, or summarization of responses was performed before the analysis.

### 2.4 Readability assessment

Readability was assessed for each response using seven established indices: Gunning Fog Index (GFI) (28), Flesch Reading Ease Score (FRES), Flesch Kincaid Grade Level (FKGL), Simple Measure of Gobbledygook (SMOG), Coleman Liau Index (CLI), Linsear Write Formula (LWF), and Automated Readability Index (ARI) (29, 30). Scores were calculated using standardized online readability calculators. Each response was evaluated independently, and the scores for all indices were recorded for every question and model. Mean values and standard deviations were calculated across the fifteen questions for each model.

### 2.5 Accuracy assessment

Three independent medical experts from the field evaluated independently for the accuracy of each response using a predefined scoring rubric with a range from 0 to 6. Higher scores showed greater factual correctness, clinical validity, and consistency with established pediatric mental health knowledge. A score of 6 indicated fully accurate and clinically sound information, while lower scores indicated partial accuracy or factual limitations. Accuracy scores were recorded at the question level for each model.

The scoring rubric was based on established clinical practice guidelines for pediatric depression, including recommendations from the National Institute for Health and Care Excellence (NICE) and the American Academy of Child and Adolescent Psychiatry (AACAP). Thus, accuracy ratings distinguished between the general factual correctness and concordance with guideline-based diagnostic criteria, risk assessment, pharmacological safety considerations, and management recommendations. Inter-rater reliability for accuracy scoring was examined by using the intraclass correlation coefficient. The coefficient demonstrated good agreement among the three experts, indicating uniformity in the scoring system.

### 2.6 Completeness assessment

Completeness was assessed separately from accuracy using the same 0 to 6 scoring scale by three medical experts. Completeness scoring measured the extent to which each response addressed all essential components of the question. A score of 6 represented comprehensive coverage, while lower scores indicated omissions or limited depth. Completeness scores were documented for each question and model.

### 2.7 Sentiment analysis

Sentiment analysis was performed to assess the emotional tone of the AI-generated responses. Quantitative sentiment scores were obtained and categorized into predefined sentiment tone classifications. Mean sentiment scores were calculated for each model for all the questions.

### 2.8 Statistical analysis

Descriptive statistics were calculated for readability, accuracy, completeness, and sentiment scores. The results were expressed as a mean with a standard deviation. One way analysis of variance was used to compare the outcomes in LLMs. Tukey *post hoc* test was applied for pairwise comparisons when overall group differences were identified. A p value <0.05 was considered statistically significant.

## 3 Results

The responses generated by the 3 LLMs were systematically analyzed for readability, sentiment, accuracy, and completeness in the fifteen standardized pediatric depression questions.

### 3.1 Readability analysis

**Table 1** summarizes the comparative evaluation of readability indices for responses generated by ChatGPT-5, Copilot-5, and DeepSeek 3.1V. One-way analysis of variance identified statistically significant group differences for the GFI, FRES, and CLI. Copilot-5 demonstrated higher GFI values, indicating greater sentence length and lexical complexity, whereas DeepSeek 3.1V recorded higher FRES, suggesting easier comprehension. Tukey *post hoc* analysis showed a significant difference between Copilot-5 and DeepSeek 3.1V for both FRES and CLI, while comparisons involving ChatGPT-5 did not reach statistical significance. No significant differences were observed among the 3 models for FKGL, SMOG, LWF, or ARI. These findings indicate measurable variability in the readability characteristics among the models, primarily driven by differences in sentence structure and lexical complexity.

**Table 1:** Comparison of readability indices among large language models, expressed as mean with standard deviation, with group differences assessed using one way ANOVA and Tukey *post hoc* analysis.

Readability parameters	ChatGPT-5	Copilot-5	DeepSeek 3.1V	P-value*	ChatGPT-5 vs Copilot-5 **	ChatGPT-5 vs DeepSeek 3.1V**	Copilot-5 vs. DeepSeek 3.1V**
Gunning FOG	11.15 (1.61)	12.62 (2.04)	11.35 (1.37)	0.0455	0.0562	0.9442	0.1117
Flesch Reading Ease Score (FRES)	49.67 (11.16)	43.87 (12.63)	54.4 (8.83)	0.0407	0.3271	0.4715	0.0316
Flesch–Kincaid Grade Level (FKGL)	10.47 (2.19)	10.66 (2.11)	9.69 (1.44)	0.3645	0.9603	0.5271	0.3715
Simple Measure of Gobbledygook (SMOG)	9.99 (1.62)	10.13 (1.52)	9.63 (1.12)	0.6185	0.9613	0.7718	0.6086
Coleman–Liau (CL) Score	13.5 (1.79)	14.27 (2.32)	12.22 (1.56)	0.0184	0.5165	0.1717	0.0145
Linsear Write (LW)	73.53 (8.79)	73 (8.01)	74.73 (5.5)	0.8141	0.9797	0.9014	0.8058
Automated Readability Index (ARI)	12.72 (2.57)	12.55 (2.36)	12.24 (1.57)	0.8302	0.9751	0.8192	0.9201

*One way ANOVA; P<0.05 considered as significant; **posthoc tukey test; All values are presented as mean (SD).

**Figure 2:**
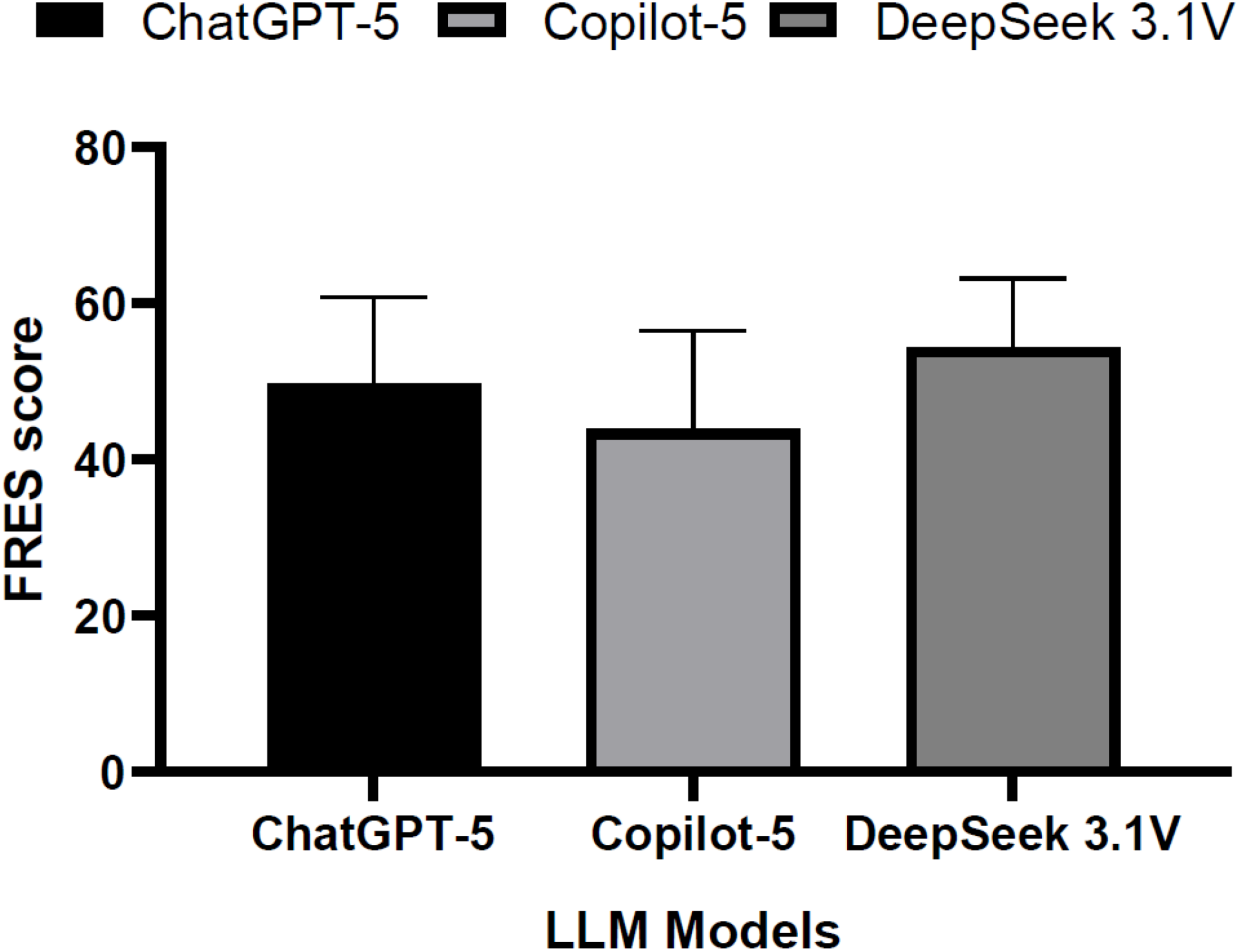
Comparison of flesch reading ease score (FRES) across ChatGPT-5, Copilot-5, and DeepSeek 3.1V for pediatric depression responses.

**Figure 2** illustrates readability patterns using the FRES, where higher values indicate easier comprehension. DeepSeek 3.1V recorded the highest mean FRES, ranging from 54 to 55, thus indicating lower language complexity among the evaluated models. ChatGPT-5 demonstrated intermediate scores of approximately 49 to 50, which corresponded to relatively easy readability. Copilot-5 recorded the lowest scores, ranging from 43 to 44. This indicated moderate reading difficulty. Error bars showed variability in responses to the fifteen questions and consistent patterns in the models.

**Figure 3:**
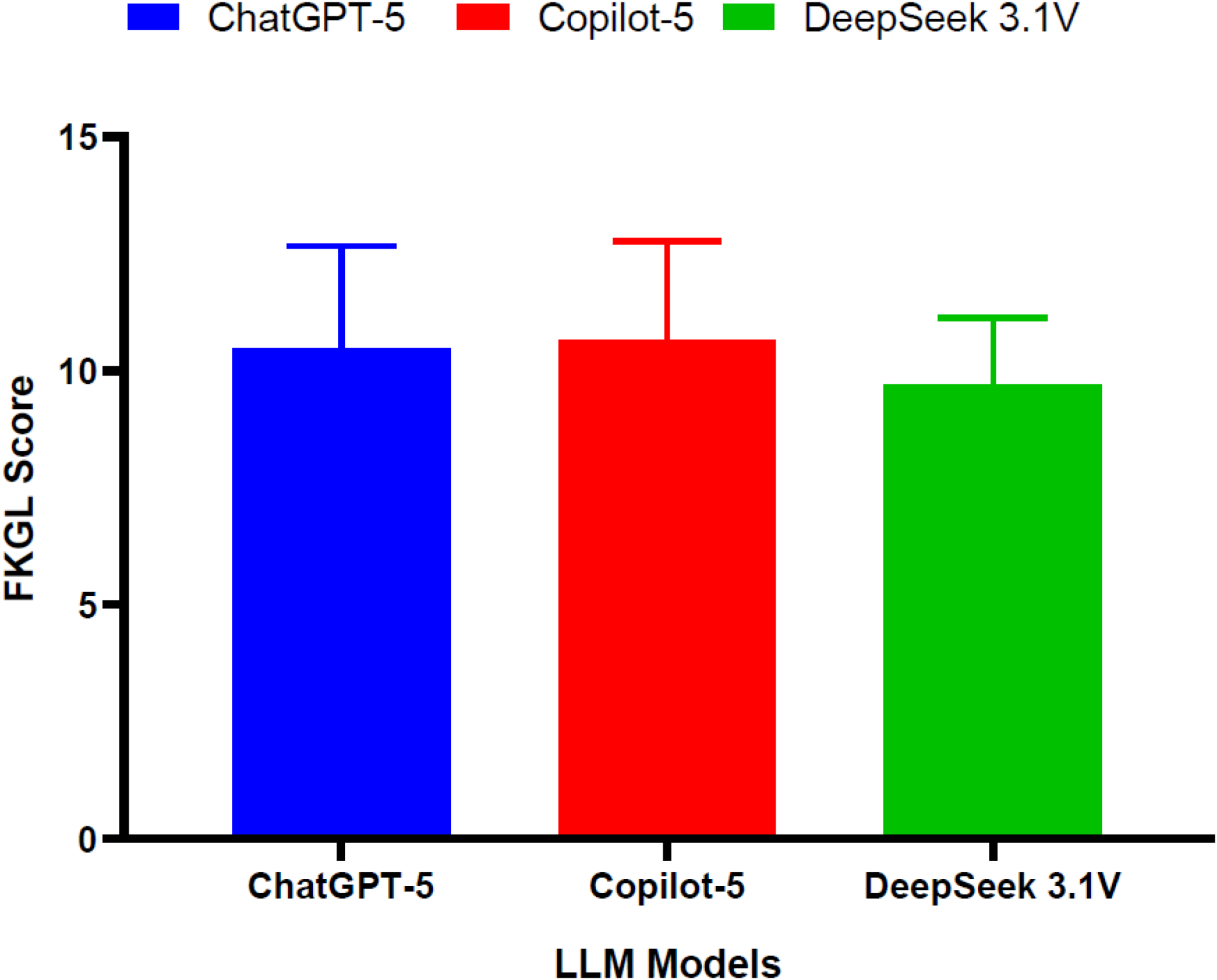
Comparison of flesch–kincaid grade level (FKGL) scores across ChatGPT-5, Copilot-5, and DeepSeek 3.1V for pediatric depression responses.

**Figure 3** presents FKGL scores for the three models. DeepSeek 3.1V recorded the lowest mean grade-level score of 9.5, corresponding to a reading level in the ninth to tenth grade range. ChatGPT-5 showed a higher mean score of approximately 10.5, while Copilot-5 recorded the highest mean score of approximately 10.8 thereby indicating a reading level at or above the eleventh grade. Variability across the questions was limited, and the relative rankings of the models were consistent. These findings was in accordance with the FRES results and indicated lower reading grade requirements for DeepSeek 3.1V responses when compared with ChatGPT-5 and Copilot-5.

### 3.2 Sentiment analysis

**Table 2** presents the comparison of sentiment scores and overall sentiment tone in the three models. ChatGPT-5 recorded the highest mean sentiment score of 63.21, followed by Copilot-5 at 42.05. DeepSeek 3.1V showed a substantially lower sentiment score of 9.09. Based on predefined sentiment classification criteria, the responses generated by ChatGPT-5 and Copilot-5 were categorized as very positive in tone, whereas DeepSeek 3.1V responses were categorized as negative. These results demonstrated marked differences in emotional tone for all the 3 models.

**Table 2:** Comparison of sentiment scores and overall sentiment tone across large language models.

Sentiment score parameters	ChatGPT-5	Copilot-5	DeepSeek 3.1V
Sentimental Score	63.21	42.05	9.09
Sentimental tone	Very Positive	Very Positive	Negative

### 3.3 Accuracy and completeness analysis

**Table 3** summarizes the comparative accuracy and completeness scores and shows that the accuracy scores performed comparably. ChatGPT-5 achieved a mean score of 4.0 ± 0.5, Copilot-5 recorded 4.1 ± 0.6, and DeepSeek 3.1V recorded 4.2 ± 0.6. One-way analysis of variance did not demonstrate a statistically significant difference among the models (p = 0.633). Tukey *post hoc* comparisons between all the model pairs also did not show statistically significant differences. Completeness scores were similarly comparable in the models. ChatGPT-5 and DeepSeek 3.1V both recorded mean completeness scores of 3.2, with standard deviations of 0.6 and 0.8, respectively, while Copilot-5 recorded a mean score of 3.3 ± 0.6. One way analysis of variance did not reveal significant group differences (p = 0.9488), and Tukey *post hoc* comparisons between all pairs were not statistically significant.

**Table 3:** Accuracy and completeness scores across large language models, expressed as mean ± standard deviation, with group differences assessed using one-way ANOVA and Tukey *post hoc* analysis.

Parameters	ChatGPT-5	Copilot-5	DeepSeek 3.1V	P-Value*	ChatGPTvs Copilot-5**	ChatGPTvs DeepSeek**	Copilot-5 vs DeepSeek**
Accuracy	4 ± 0.5	4.1 ± 0.6	4.2 ± 0.6	0.633	0.8048	0.6157	0.9469
Completeness	3.2 ± 0.6	3.3 ± 0.6	3.2 ± 0.8	0.9488	0.9575	>0.9999	0.9575

*One way ANOVA; P<0.05 considered as significant; **posthoc tukey test; All values are presented as mean (SD).

**Figure 4:**
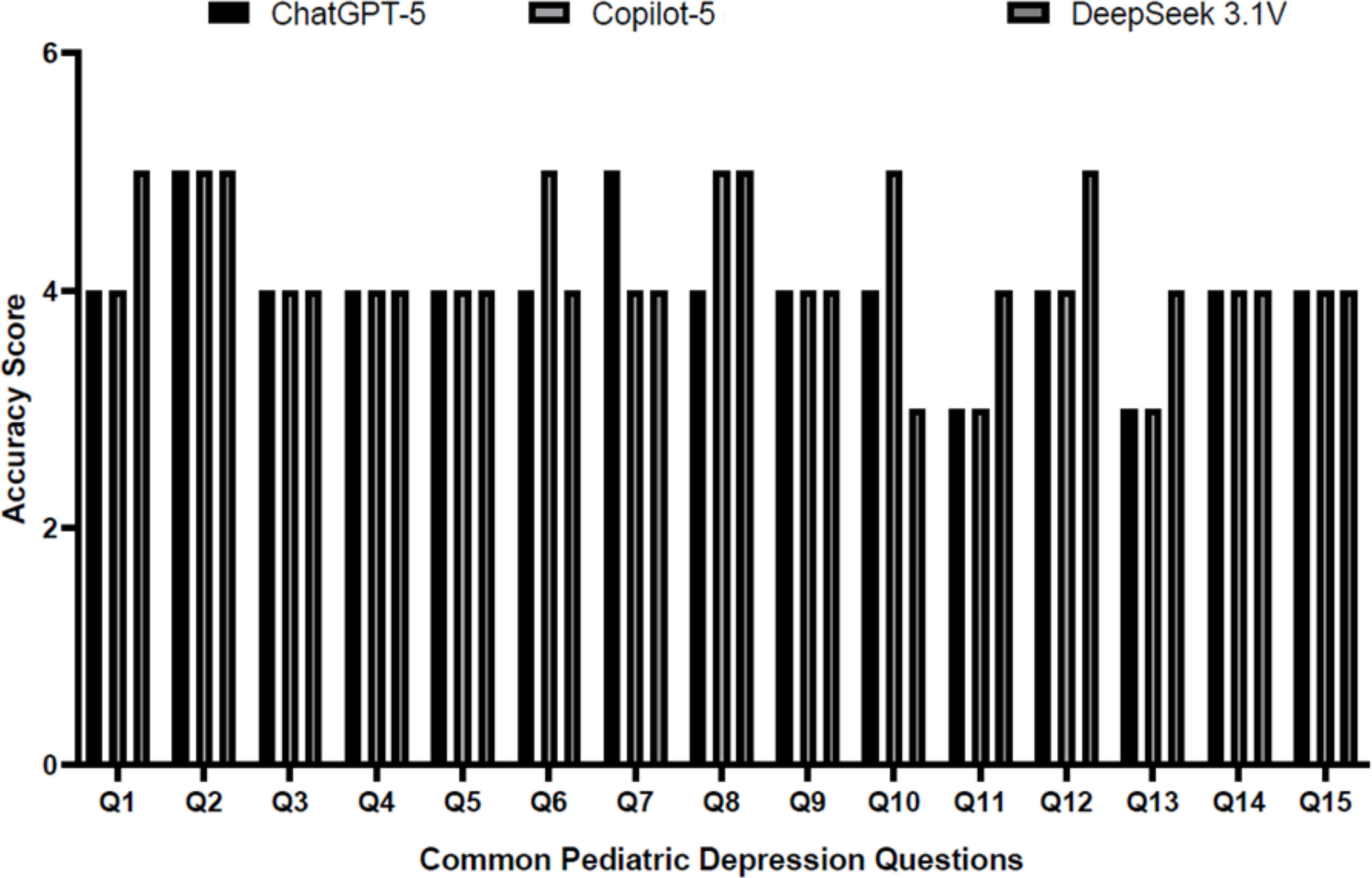
Comparison of accuracy scores (0–6 scale) for ChatGPT-5, Copilot-5, and DeepSeek 3.1V across fifteen pediatric depression queries.

**Figure 4** provides a question-level comparison of accuracy scores in the questions. Copilot-5 consistently achieved the highest or near-highest accuracy scores across most questions, frequently scoring 5 or 6, particularly for questions Q2, Q6, Q7, Q8, Q9, Q12, and Q15. ChatGPT-5 recorded scores predominantly in the 4 to 5 range, with lower scores observed for Q10, Q11, Q13, and Q14, thus indicating reduced accuracy or limited clinical detail for these queries. DeepSeek 3.1V showed a pattern similar to ChatGPT-5, with lower scores for the same clinically detailed questions. Copilot-5 demonstrated more constant accuracy in both general and clinically complex questions.

**Figure 5:**
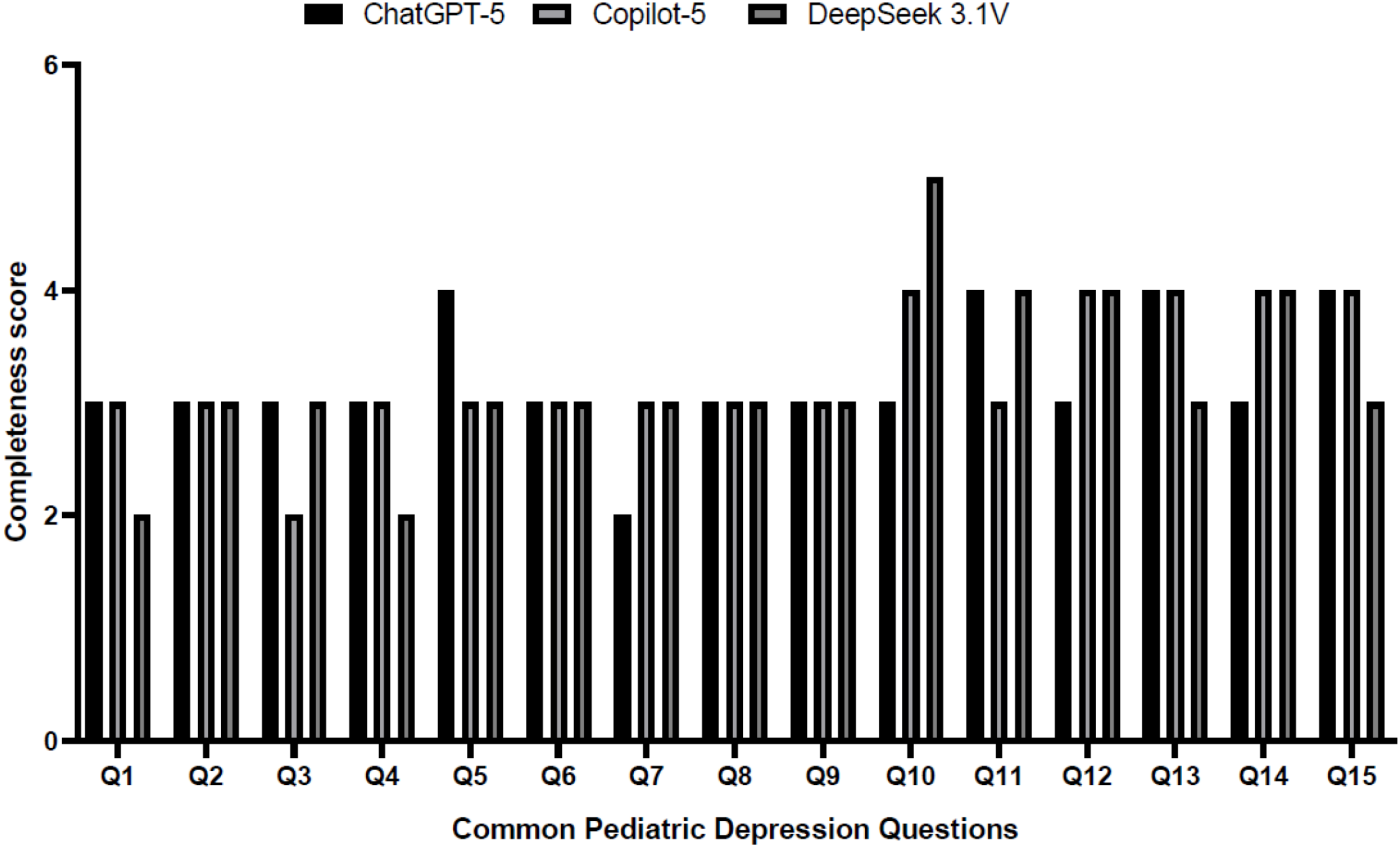
ChatGPT-5 scored highest (~5.8 on Q10) across 15 pediatric depression queries; Copilot-5 and DeepSeek 3.1V showed more variable, often lower scores.

**Figure 5** presents the completeness scores at the question level. ChatGPT-5 consistently achieved higher completeness scores across most questions, with values largely in the 4 to 5 range. The highest completeness score was observed for Q10, with a mean value of approximately 5.8, indicating extensive topic coverage. Higher completeness scores were also noted for Q5, Q7, and Q11. Copilot-5 showed greater variability in completeness, with lower scores for selected questions requiring detailed clinical explanations. DeepSeek 3.1V demonstrated the widest variability, with lower completeness scores for several clinically focused questions. ChatGPT-5 provided more comprehensive responses across multiple questions, while Copilot-5 and DeepSeek 3.1V showed greater inconsistency in completeness.

## 4 Discussion

The use of LLMs as informal sources of medical information has increased, with patients and caregivers frequently consulting these tools for complex topics like pediatric depression. Families who notice mood changes OR irritability in their child may depend upon accessible sources such as LLMs for guidance, which can influence whether and how quickly they seek professional help. LLMs show promising results in healthcare communication but their outputs can vary significantly in quality, accuracy, and reliability thus developing concerns for susceptible populations (10, 27, 31). In pediatric depression, symptoms are often mistaken for normal adolescent behavior, and clear, accurate, and emotionally supportive information may help reduce delays in seeking appropriate care. More than 60% of parents first search online before contacting a clinician about behavioral changes in their child (7), and adolescents aged 12 to 17 are among the highest users of digital mental health tools (21). The quality of LLM responses therefore affects early recognition and help seeking in pediatric depression Therefore, structured, multi-dimensional assessments are needed to evaluate their performance in specific clinical contexts. This cross-sectional study evaluated the readability, accuracy, completeness, and sentiment of responses generated by three advanced LLMs to frequently asked questions related to pediatric depression.

Readability analysis showed differences across the evaluated models. DeepSeek 3.1V generated responses with higher FRES values of approximately 54 to 55 and lower FKGL scores around 9.5, indicating text suitable for early high school readers. ChatGPT-5 showed moderate readability, while Copilot-5 produced more complex language, as evidenced by lower FRES values (43–44) and higher FKGL scores (10.8). These patterns depict a variation in the length of the sentence and choice of the words in various models. Ozduran et al. (16) assessed AI generated information on low back pain and reported FKGL values between 11.2 and 12.9 in various platforms. These values were similar to the grade level scores observed for ChatGPT-5, Copilot-5 (Smart Research), used in the present analysis. Gondode et al. (13) evaluated the chatbot generated content in ophthalmic anesthesia and reported FKGL scores of 11.7 for ChatGPT and 10.1 for Gemini, which was more than the commonly cited eighth grade level for patient information. Güldiken et al. (12) examined English language responses from ChatGPT-4.5 with relation to orthognathic surgery and reported a FRES of 48.4 and an FKGL of 10.5. This was in accordance to the readability profile of ChatGPT-5 used in the present study. These authors also reported a higher readability for DeepSeek-V3-R1, with a FRES of 52.1 and an FKGL of 9.8. Similarly, DeepSeek 3.1V produced text with higher FRES values and lower FKGL scores in this study. This is important in pediatric depression because the average health literacy of U.S. caregivers is at an 8^th^ to 9^th^ grade level (30), and adolescents with early depression often have difficulty with concentration and processing of the information (5). FKGL score of 10.8 was observed for Copilot-5 responses, which could limit the understanding of information related to antidepressant safety and warning signs of suicidality for many caregivers. Therefore, these findings indicate that AI-generated medical information frequently requires reading skills above the recommended levels. This is more relevant to pediatric mental health, where adolescents and caregivers may have different levels of literacy. Abeo et al. (8) reported that automated text generation often depends upon the technical phrasing, which can limit the comprehension capacity of the readers with lower health literacy levels. The present results show that readability varies in all the models thus indicating that language complexity is dependent upon the design of the model and training and is not uniform among different systems.

In the present study, the accuracy scores in all three models were generally high, with mean values >4 on a six-point scale. Copilot-5 showed the most stable outcomes, especially for questions related to diagnosis and management, where several items scored between 5 and 6. Hanss et al. (23) conducted a study using GPT-4 and Claude 2 and achieved accuracy rates of 85% and 84% on standardized psychiatry multiple-choice questions, which were higher than GPT-3.5’s performance (68%). In the present study, the results observed for ChatGPT-5, Copilot-5 (Smart Research), were similar to these observations thus suggesting a pattern of improvement across newer model versions. Xu et al. (20) reported an accuracy >80% for general psychiatry knowledge tasks, with lower performance for diagnostic reasoning and management decisions. Neubauer et al. (24) reported similar results in child and adolescent psychiatry, where ChatGPT-4 and Gemini Pro achieved accuracy levels of 79% and 68%. The performance was higher for structured knowledge domains and lower for clinically complex topics. The authors observed that no model exceeded 80% accuracy on child and adolescent psychiatry board style questions thus depicting a similar upper limit to performance. Similarly, in the present study, Copilot-5 did not reach a perfect mean score. In the present study, all three models showed lower accuracy for questions addressing pharmacotherapy, comorbid conditions, and prognosis, with scores reducing to approximately 2.5 to 3.2 for selected items. In response to Q10 *(“What are the long-term effects of untreated depression in teens?”)*, only ChatGPT-5 showed a 2-to-3-fold increased risk of depression recurrence in adulthood and the risk of substance use disorders, which are relevant for decision making capacity of the caregiver (1, 6). When asked about SSRI risks in adolescents (Q12), Copilot-5 mentioned the FDA black box warning in 80% of responses, while DeepSeek 3.1V did not mention this warning in most of the responses, which may restrict the understanding of the caregiver about medication safety. Li et al. (18) reported comparable patterns in pediatric fracture management, where accuracy decreased for questions related to surgical decision making and complication management. Sezgin et al. (19) reported similar limitations in responses to postpartum depression queries, with high accuracy for symptom recognition but omission of guideline-based elements in a proportion of responses. Douma et al. (31) reviewed pediatric applications of LLMs and reported variable accuracy for treatment related content. Hence, these findings show that LLMs are capable to monitor the core psychiatric concepts dependably but have difficulty with detailed clinical decisions that require guideline interpretation and developmental background.

Completeness scores varied prominently in all three evaluated LL models. ChatGPT-5 produced responses that were related to a wider range of clinical elements in all fifteen questions, with higher scores for several treatment-focused items. Copilot-5 and DeepSeek 3.1V showed greater distribution in scores, and several responses removed the relevant sub-components, thus resulting in lower completeness ratings. Similarly, Gondode et al. (13) showed that professionally prepared patient information leaflets in ophthalmic surgery covered more required elements than any chatbot-generated text. Lieu et al. (15) reported that ChatGPT-4 responses to pediatric scoliosis questions fulfilled the predefined clinical completeness criteria for a large proportion of items therefore suggesting that structured question formats probably produce better inclusion. In child and adolescent psychiatry, Neubauer et al. (24) reported that model responses often did not include differential diagnoses or long-term management elements. A caregiver searching for information about child irritability may receive a response that recognizes depression as a possible cause but does not mention about the common comorbid conditions like attention-deficit/hyperactivity disorder or anxiety, which occur in 40 to 60% of pediatric cases (4, 6). This may affect the caregiver’s decision to seek a comprehensive clinical evaluation. In this study, Copilot-5 and DeepSeek 3.1V responses frequently removed the discussion of prognosis and comorbid conditions. Variation of findings between the models was related to the differences in response organization and level of detail. ChatGPT-5 more frequently included the clinical information with psychosocial factors and follow-up considerations in a single response. Copilot-5 often generated brief answers, which restricted the insertion of contextual and explanatory details. Xu et al. (20) reported a comparable observation in mental health tasks, where shorter responses had fewer explanatory components. These present findings show that the extent of topic coverage varies by the language model and type of the questions. This variation supports the need for critical appraisal when automated content is used for pediatric mental health information, especially for questions that require discussion in multiple clinical domains.

Sentiment analysis showed clear differences in the emotional tone of the 3 LLMs used in this study. ChatGPT-5 and Copilot-5 primarily produced responses classified as very positive, while DeepSeek 3.1V more often generated responses classified as negative. Such differences in sentiment analysis have also been reported by Gondode et al. (13), who observed a negative compound sentiment score for ChatGPT and a positive score for Gemini in ophthalmic patient education content thus indicating that emotional tone varies by model design rather than by clinical topic. The emotional tone carries specific significance in mental health communication. Adolescents with depression show higher rates of disengagement from digital mental health content that uses neutral or clinical wording (22). In this group, 73% cases report an increased sensitivity to criticism or negatively framed information, and neutral or clinical wording may be perceived as dismissive or stigmatizing (22). Alanezi et al. (25) reported that users appreciated emotionally thereby validating and encouraging the language in ChatGPT based mental health interactions, with a large proportion of participants identifying the tone as an important factor for trust. Shin et al. (22) showed that emotionally neutral or negative word or phrase in adolescent depression screening reduced the engagement and perceived usefulness. Gu et al. (26) demonstrated that choice of word alone, such as the use of terms describing concern rather than symptoms, were efficient to change the emotional perception of depression related tasks even when factual content was unaffected. In the present study, the more negative sentiment profile was observed for DeepSeek 3.1V which depicts a cautious and risk focused style of expression. In response to the question *“Is my child’s irritability a sign of depression?”*, DeepSeek 3.1V used terms like “*serious condition,” “high risk of deterioration,”* and *“urgent psychiatric evaluation needed”* without stating that pediatric depression is treatable. This type of phrases might increase the anxiety of the caregiver and affect the timely help seeking capacity (5, 25). The anxiety of the caregiver has been reported as a barrier to mental health service use in 38% of the families of adolescents with depression (7). Levkovich et al. (32) reported a similar propensity in childhood anxiety scenarios, where AI systems highlighted the severity and complexity strongly than the clinicians. In contrast, ChatGPT-5 responses adopted the supportive phrasing more while presenting the specific clinical information. Hence, these findings indicate that the emotional tone differs in all the LLMs and may have an impact on how the pediatric mental health information is received. Models that emphasize on caution may increase the pain, while those that favor the supportive language may promote comfort. Therefore, it is essentially relevant to consider emotional tone when automated responses are used for pediatric mental health queries.

DeepSeek 3.1V produced responses with easy language but a more negative emotional tone, while ChatGPT-5 combined moderate readability with positive words. Abeo et al. (8) reported that clear language is insufficient when emotional framing is inappropriate, since the tone can affect the understanding capacity and user response. Copilot-5 showed a positive tone but provided less complete information thus indicating that emotional wording does not confirm adequate content coverage. Similar findings were reported in oncology education, where AI generated material showed favorable sentiment while removing the important clinical details (9). This study assessed readability, accuracy, completeness, and sentiment of responses to standardized pediatric depression questions. Previous studies have focused on the accuracy or automated scoring primarily and did not measure the emotional tone or the extent of content coverage (23, 24). Accuracy alone does not assure that automated medical information is suitable for pediatric populations (31). It has been reported that one in three adolescents with depression do not receive treatment, often because the symptoms are misdiagnosed or caregivers are uncertain about the need for care (1, 6). The responses of the LLM that use clear language, provide complete clinical information, and while maintaining a supportive tone, may support good engagement with mental health services. It is estimated that 52% of parents scheduled a mental health evaluation for their child after reading online health information that used supportive and non-alarming language (7). The present study evaluates various dimensions within a pediatric framework to examine how LLMs present clinically relevant mental health information in such pediatric cases.

Wang et al. (17) reported a higher completeness score for ChatGPT when compared with Bard and Claude in myopia prevention guidance, while readability remained limited in various models. Yigit et al. (14) observed omissions in AI-generated pediatric asthma management content, especially for dose and long-term care. Güldiken et al. (12) reported similar variability in orthognathic surgery education, with the performance being influenced by the language and structure. These examples indicate that the features identified in pediatric depression responses shows broader patterns of LLM behavior in child health information delivery. ChatGPT-5 provided more complete, positive responses; Copilot-5 showed constant accuracy, and DeepSeek 3.1V used easy language throughout. These differences represent the model architecture, training data, and output optimization on response characteristics (12, 14, 15). The observed variation highlights the necessity for careful, domain-specific evaluation before using such LLMs for pediatric mental health information wherein any incomplete or poorly framed content may lead to misunderstanding or confusion (7, 33). In pediatric depression, early intervention reduces the risk of recurrence in adulthood by approximately 50% (6). The differences in the manner LLMs describes the risk, treatment options, and recommended actions may influence the long-term outcomes. The average delay between symptom onset and treatment initiation is 2 to 3 years (1, 5), and LLMs that provide timely, accurate, and emotionally appropriate information which could help to reduce this delay.

A Pediatric Artificial Intelligence Safety Framework can reduce risks when large language models are used in pediatric mental health by including four essential checkpoints that guide safe clinical use. Pharmacological recommendations should not be provided without clinician supervision. Responses which discuss the selective serotonin reuptake inhibitors must clearly state black box warnings, as error in safety information may affect the appropriate decision making (34, 35). Readability should resemble grade 8 health literacy levels approximately, and language should be supportive to reduce the anxiety of the caregiver (36). Any mention of self-harm or suicidality must include clear instructions for immediate professional care and provide verified crisis resources, since AI systems may fail to highlight the high-risk situations properly. A clinician’s review is required before AI generated outputs inform the caregiver decisions (34–36). Hence, this framework facilitates safe informational use in concordance with pediatric clinical standards.

## 5 Strengths and limitations

A comprehensive evaluation included various aspects of the model performance to provide a detailed profile of each LLM. The strengths of this study include the use of validated scoring tools and structured analysis, which allowed a systematic comparison among the clinically relevant queries. The limitations included the cross-sectional design conducted at a single time point and the use of a single-response sampling approach for each question, which may not capture the variability in repeated model outputs. Subjective expert scoring, limitations of readability and sentiment measures in evaluating full comprehension or clinical suitability, and the restricted scope of the standardized frequently asked questions were additional drawbacks.

## 6 Conclusion

Contemporary LLMs provide diverse information on pediatric depression, with each model demonstrating distinct advantages and limitations. DeepSeek 3.1V produces responses with the most accessible language, Microsoft Copilot GPT-5 (Smart Research) shows the highest factual consistency, and ChatGPT-5 delivers the most complete coverage. All these models show elevated reading levels, extensive content, and variable emotional tone, and are best positioned as informational support tools and health literacy aids that may provide preliminary guidance or digital triage support, but not diagnostic or treatment decisions. Thus, these LLMs cannot replace any professional guidance in patient education. A clinician should review and contextualize any AI-generated responses before parents or caregivers use them. Future research should focus on refining models for pediatric health literacy, establishing standardized mental health content, and assessing their impact on help-seeking behavior.

## Data Availability

The original contributions presented in the study are included in the article/**Supplementary Material**. Further inquiries can be directed to the corresponding author.
