# Supplementary material for: Assessing large language model responses to pediatric depression FAQs: a cross-sectional study on readability, accuracy, and sentiment
Source: Front Psychiatry. 2026 Apr 29;17:1782288. doi: 10.3389/fpsyt.2026.1782288 (PMC13168101; doi:10.3389/fpsyt.2026.1782288)
Supplement: Supplementary file 1 [file Table1.docx]

**Supplementary Table 1:** List of common patient questions

| Q1: | What is depression in children? |
| --- | --- |
| Q2: | What are the types of depression and mood disorders in children? |
| Q3: | What are risk factors for childhood depression? |
| Q4: | What causes depression in children? |
| Q5: | How do I know if my child is depressed? |
| Q6: | How can I help my child with depression? |
| Q7: | Does my child need counseling for depression symptoms? |
| Q8: | What treatment strategies would be useful for my children with depression? |
| Q9: | How is depression diagnosed in children? |
| Q10: | Can I prevent my child from developing depression? |
| Q11: | What can I expect if my child has depression? |
| Q12: | What are the advanced treatments currently available for pediatric depression? |
| Q13: | Will the pediatric depression continue when the child became adult? |
| Q14: | How would I know if my child's depression is gone? |
| Q15: | Does my child's depression impact their education level or employability? |

**Supplementary Table 2:** Response from different AI chatbots

**Response from Copilot (Model Name: SMART GPT-5)**

**Q1 What is depression in children?**

**Response:**

**🌱 What is Depression in Children?**

**It’s normal for children to feel sad, upset, or irritable from time to time. But depression is more than ordinary sadness. Depression in children is a medical condition where these feelings are strong, last for weeks or longer, and interfere with daily life—such as school, friendships, family relationships, or play.**

**Key Points for Caregivers**

- **Not just a phase: Depression is not simply “moodiness” or something a child can “snap out of.”**
- **How it shows up: Children may seem sad, angry, or withdrawn. They may lose interest in activities they used to enjoy, struggle with sleep or appetite, or have trouble concentrating.**
- **Impact on daily life: Depression can make it hard for a child to keep up with schoolwork, friendships, or family routines.**
- **Causes: There’s no single cause. It can be linked to a mix of factors—family history, stressful life events, bullying, or even changes in brain chemistry.**
- **It is treatable: With the right support—such as counseling, family involvement, and sometimes medication—children can and do get better.**

**Why Early Help Matters**

**Just like you would take a child to the doctor for a broken arm, seeking help early for depression is important. Early treatment can prevent symptoms from getting worse and support healthy emotional development.**

**👉 If you’re a caregiver, the most important thing you can do is listen, take your child’s feelings seriously, and reach out for professional support. Depression is treatable, and children can recover and thrive with the right care.**

**Q2 What are the types of depression and mood disorders in children?**

**Response:**

**Types of Depression and Mood Disorders in Children**

**Children and teens can experience different kinds of mood disorders. These conditions affect how they feel, think, and act—sometimes for weeks or months at a time. Understanding the types can help caregivers recognize when to seek help.**

**1. Major Depressive Disorder (MDD)**

- **Strong, ongoing sadness or irritability.**
- **Loss of interest in activities they once enjoyed.**
- **Changes in sleep, appetite, energy, or concentration.**
- **Symptoms last at least two weeks and interfere with daily life.**

**2. Persistent Depressive Disorder (Dysthymia)**

- **A milder but longer‑lasting form of depression.**
- **Children may seem “down” or irritable most of the time for a year or more.**
- **Can affect self‑esteem, school performance, and friendships.**

**3. Bipolar Disorder**

- **Involves mood swings between extreme highs (mania or hypomania) and lows (depression).**
- **During highs: children may seem unusually energetic, talkative, or need little sleep.**
- **During lows: symptoms look similar to depression.**

**4. Disruptive Mood Dysregulation Disorder (DMDD)**

- **Frequent, intense temper outbursts (verbal or physical).**
- **Irritable or angry mood most of the day, nearly every day.**
- **Symptoms usually begin before age 10.**

**5. Adjustment Disorder with Depressed Mood**

- **Sadness or irritability triggered by a stressful life event (e.g., moving, divorce, loss).**
- **Symptoms appear within a few months of the event and usually improve with support.**

**6. Other Mood‑Related Conditions**

- **Seasonal Affective Disorder (SAD): Depression linked to changes in seasons, often winter.**
- **Premenstrual Dysphoric Disorder (PMDD): Severe mood symptoms in adolescent girls before menstruation.**

**Why This Matters**

- **These conditions are not just phases—they are medical issues that deserve attention.**
- **Early recognition and treatment (therapy, family support, sometimes medication) can make a big difference.**
- **Caregivers play a vital role by noticing changes, offering support, and seeking professional help.**

**Q3 What are risk factors for childhood depression?**

**Response:**

**🌱 Risk Factors for Childhood Depression**

**Depression in children does not have a single cause. Instead, it often develops from a combination of biological, psychological, and environmental factors. Understanding these risk factors can help caregivers recognize when a child may need extra support.**

**🧬 Biological and Family Factors**

- **Family history of depression or other mental health conditions**
- **Imbalances in brain chemistry that affect mood regulation**
- **Chronic medical conditions (e.g., diabetes, asthma, epilepsy)**

**🏠 Environmental and Social Factors**

- **Stressful or traumatic life events such as divorce, loss of a loved one, or abuse**
- **Bullying or peer rejection at school or online**
- **Family conflict or lack of emotional support at home**
- **Poverty or financial stress that creates ongoing instability**

**💭 Psychological and Developmental Factors**

- **Low self-esteem or feelings of worthlessness**
- **Difficulty coping with stress or problem-solving**
- **Other mental health conditions, such as anxiety or ADHD, which can increase vulnerability**

**💡 Why This Matters**

- **Having one or more of these risk factors does not mean a child will definitely develop depression.**
- **It does mean the child may be more vulnerable, so early recognition and support are especially important.**
- **Protective factors—such as strong family bonds, supportive friendships, healthy routines, and access to care—can reduce risk and help children build resilience.**

**👉 Takeaway for caregivers: If your child is facing several of these challenges and shows ongoing sadness, irritability, or withdrawal, it’s important to seek professional guidance. With the right support, children can recover and thrive.**

**Q4 What causes depression in children?**

**Response:**

**Depression in children does not come from a single cause. Instead, it usually develops from a combination of factors—biological, psychological, and environmental—that interact in complex ways.**

**🧬 Biological Factors**

- **Family history: Children with parents or close relatives who have depression or other mental health conditions are at higher risk.**
- **Brain chemistry: Imbalances in neurotransmitters (the brain’s “messenger chemicals”) can affect mood regulation.**
- **Medical conditions: Chronic illnesses such as asthma, diabetes, or epilepsy can increase vulnerability.**

**💭 Psychological Factors**

- **Low self-esteem or negative self-image**
- **Difficulty coping with stress or problem-solving**
- **History of trauma or abuse (emotional, physical, or sexual)**

**🏠 Environmental and Social Factors**

- **Stressful life events such as divorce, loss of a loved one, or moving to a new place**
- **Bullying, peer rejection, or social isolation**
- **Family conflict or lack of emotional support at home**
- **Academic pressure or ongoing struggles at school**

**💡 Important to Remember**

- **Depression is not caused by weakness, laziness, or “bad behavior.”**
- **Having risk factors does not mean a child will definitely develop depression—but it does mean they may need extra support.**
- **Protective factors—like strong family bonds, supportive friendships, healthy routines, and access to care—can help children build resilience.**

**👉 Takeaway for caregivers: Depression in children is usually the result of several overlapping influences, not one single cause. If your child shows ongoing sadness, irritability, or withdrawal, reaching out to a healthcare professional early can make a big difference.**

**Q5 How do I know if my child is depressed?**

**Response:**

**It’s normal for children to feel sad, upset, or irritable at times. But depression is different—it lasts longer, feels stronger, and interferes with daily life. Caregivers often notice changes in their child’s emotions, behavior, or physical health that don’t seem to go away.**

**🧩 Common Signs to Watch For**

- **Mood changes: Persistent sadness, irritability, anger, or frequent crying.**
- **Loss of interest: No longer enjoying activities, play, or hobbies they once liked.**
- **Social withdrawal: Avoiding friends, family, or preferring to be alone.**
- **School difficulties: Drop in grades, trouble concentrating, or loss of motivation.**
- **Changes in sleep or appetite: Sleeping too much or too little, eating much more or less than usual.**
- **Low energy: Seeming tired, sluggish, or lacking enthusiasm.**
- **Feelings of hopelessness or worthlessness: Expressing negative thoughts about themselves or the future.**
- **Physical complaints: Frequent headaches, stomachaches, or other pains without a clear medical cause.**

**🚩 When to Seek Help Immediately**

- **If your child talks about wanting to die, self‑harm, or feeling like life isn’t worth living, seek professional help right away.**

**💡 Important to Remember**

- **Depression is not just a phase or something a child can “snap out of.”**
- **Having some of these signs doesn’t always mean depression, but if they last for more than two weeks or interfere with daily life, it’s time to reach out for support.**
- **Early help makes a big difference—just like treating a physical illness early.**

**👉 Takeaway for caregivers: Trust your instincts. If your child’s mood or behavior feels “off” for a long time, or you’re worried, it’s best to talk with a pediatrician or mental health professional. With the right care, children can and do get better.**

**Q6 How can I help my child with depression?**

**Response:**

**Caring for a child with depression can feel overwhelming, but your support is one of the most powerful tools in their recovery. While professional treatment is important, there are many meaningful ways you can help at home.**

**🧩 Be Present and Listen**

- **Create a safe space where your child feels heard.**
- **Listen without judgment or rushing to “fix” things.**
- **Reassure them that their feelings are real and important.**

**🏠 Provide Stability and Routine**

- **Keep regular schedules for meals, sleep, school, and activities.**
- **Predictable routines help children feel secure and reduce stress.**

**🌱 Encourage Healthy Habits**

- **Support good sleep, balanced nutrition, and regular physical activity.**
- **Encourage creative outlets like drawing, music, or journaling.**
- **Limit screen time if it interferes with sleep or mood.**

**💬 Talk Openly About Mental Health**

- **Explain that depression is a medical condition, not their fault.**
- **Normalize asking for help—just like with a physical illness.**

**🤝 Seek Professional Support**

- **Reach out to your pediatrician, school counselor, or a mental health professional.**
- **Therapy, family involvement, and sometimes medication may be part of treatment.**
- **Stay engaged in their care and encourage consistency with treatment.**

**🚨 Know When It’s an Emergency**

- **If your child talks about wanting to hurt themselves or not wanting to live, take it seriously.**
- **Stay with them and seek immediate help by calling emergency services or the 988 Suicide & Crisis Lifeline (U.S.).**

**💡 Care for Yourself Too**

- **Supporting a child with depression can be emotionally draining.**
- **Make time for your own rest, support networks, and self‑care—you’ll be stronger for your child.**

**👉 Takeaway for caregivers: Your love, patience, and advocacy are vital. Depression is treatable, and with professional care plus your steady support, children can recover and thrive.**

**Q7 Does my child need counseling for depression symptoms?**

**Response:**

**It’s natural for children to feel sad, worried, or irritable at times. But when these feelings are strong, last for weeks, and interfere with daily life, it may be a sign of depression—and counseling can help.**

**🧩 When Counseling May Be Needed**

**Consider reaching out to a mental health professional if your child:**

- **Shows persistent sadness, irritability, or anger lasting more than two weeks.**
- **Withdraws from friends, family, or activities they used to enjoy.**
- **Has changes in sleep or appetite that don’t improve.**
- **Struggles with school performance, focus, or motivation.**
- **Complains of frequent headaches, stomachaches, or fatigue without a clear medical cause.**
- **Expresses feelings of worthlessness, hopelessness, or guilt.**
- **Talks about self-harm or not wanting to live (this is an emergency—seek immediate help).**

**🌱 How Counseling Helps**

- **Provides a safe space for your child to express feelings.**
- **Teaches healthy coping skills for stress, sadness, and anxiety.**
- **Helps families learn ways to support their child at home.**
- **Can be combined with medical treatment if needed.**

**💡 Important to Remember**

- **Counseling is not a sign of failure—it’s a step toward healing.**
- **Early support can prevent symptoms from worsening and help your child build resilience.**
- **Even if you’re unsure, talking with your pediatrician or a child therapist can give clarity and peace of mind.**

**👉 Takeaway for caregivers: If your child’s mood or behavior has changed in ways that worry you, it’s better to seek help early. Counseling can provide tools, support, and hope—for both your child and your family.**

**Q8 What treatment strategies would be useful for my children with depression?**

**Response:**

**Depression in children is treatable. The most effective approach usually combines professional care with support at home. Every child is unique, so treatment plans are tailored to their needs.**

**🧩 Professional Treatment Options**

- **Counseling/Therapy**
  - ***Cognitive Behavioral Therapy (CBT):* Helps children recognize negative thought patterns and build healthier coping skills.**
  - ***Play therapy or talk therapy:* Provides a safe space for younger children to express feelings.**
  - ***Family therapy:* Strengthens communication and support within the family.**
- **Medication (when recommended)**
  - **In some cases, doctors may prescribe antidepressants.**
  - **Medication is usually combined with therapy and closely monitored by a pediatrician or child psychiatrist.**
- **School-based support**
  - **Collaboration with teachers and counselors can help with academic adjustments, peer support, and stress management.**

**🌱 Supportive Strategies at Home**

- **Build routine: Regular sleep, meals, and activities create stability.**
- **Encourage healthy habits: Balanced nutrition, physical activity, and outdoor time can improve mood.**
- **Positive communication: Offer encouragement, listen without judgment, and validate their feelings.**
- **Limit stressors: Reduce unnecessary pressures and help your child manage school or social challenges.**
- **Model coping skills: Show healthy ways of handling stress—children often learn by watching caregivers.**

**🚨 Crisis Support**

- **If your child talks about self-harm or not wanting to live, treat it as an emergency.**
- **Stay with them and seek immediate help by calling emergency services or the 988 Suicide & Crisis Lifeline (U.S.) or your local crisis helpline.**

**💡 Takeaway for Caregivers**

**Depression is not a sign of weakness—it’s a medical condition that responds to treatment. With professional care, family support, and healthy daily habits, children can recover and thrive.**

**Q9 How is depression diagnosed in children?**

**Response:**

**Depression in children is not diagnosed with a single test, like a blood test or scan. Instead, doctors and mental health professionals use a careful evaluation process to understand your child’s symptoms, experiences, and overall health.**

**🔍 Steps in the Diagnostic Process**

- **Medical check‑up: A pediatrician may start with a physical exam and lab tests to rule out medical conditions (like thyroid problems or vitamin deficiencies) that can mimic depression.**
- **Detailed history: The doctor or mental health professional will ask about your child’s mood, behavior, sleep, appetite, school performance, friendships, and family life.**
- **Interviews and questionnaires: Your child, you, and sometimes teachers may be asked to complete questionnaires or rating scales about symptoms and daily functioning.**
- **Observation: Clinicians may observe how your child interacts, expresses emotions, and responds during the visit.**
- **DSM‑5 criteria: Professionals use guidelines from the *Diagnostic and Statistical Manual of Mental Disorders (DSM‑5)* to determine if your child’s symptoms meet the criteria for depression.**

**🧠 Who Makes the Diagnosis?**

- **Pediatricians often begin the process and may refer to specialists.**
- **Child psychiatrists, psychologists, or licensed therapists are trained to make a formal diagnosis and recommend treatment.**

**💡 Important to Remember**

- **Occasional sadness or irritability is normal. A diagnosis is considered only when symptoms are persistent (lasting at least two weeks), severe, and interfering with daily life.**
- **Getting an accurate diagnosis is the first step toward effective treatment and recovery.**

**👉 Takeaway for caregivers: If your child shows ongoing sadness, irritability, or withdrawal that doesn’t improve, talk with your pediatrician or a mental health professional. Early diagnosis and treatment can make a big difference in your child’s well‑being.**

**Q10 Can I prevent my child from developing depression?**

**Response:**

**Depression cannot always be completely prevented—some children may develop it due to genetics, brain chemistry, or life events outside of anyone’s control. However, caregivers play a powerful role in lowering risk and building resilience. By creating a supportive environment and teaching healthy coping skills, you can help protect your child’s mental well‑being.**

**🏠 Build a Supportive Home Environment**

- **Show consistent love, attention, and encouragement.**
- **Keep open communication—let your child know it’s safe to share feelings.**
- **Model healthy ways of handling stress and emotions.**

**🌱 Encourage Healthy Habits**

- **Maintain regular routines for meals, sleep, and school.**
- **Support physical activity and outdoor play, which boost mood.**
- **Encourage balanced nutrition and limit excessive screen time.**

**💬 Teach Coping and Problem‑Solving Skills**

- **Help your child learn to manage stress in positive ways (deep breathing, journaling, creative activities).**
- **Praise effort and progress, not just results, to build self‑esteem.**
- **Teach them that it’s okay to ask for help when things feel overwhelming.**

**🤝 Strengthen Social Connections**

- **Encourage friendships and positive peer activities.**
- **Stay involved in your child’s school life and community.**
- **Help them feel connected to supportive adults beyond the family (teachers, mentors, relatives).**

**🔍 Watch for Early Signs**

- **Be alert to changes in mood, sleep, appetite, or school performance.**
- **Seek professional guidance early if you notice persistent sadness, irritability, or withdrawal.**

**💡 Takeaway for Caregivers**

**You cannot guarantee that your child will never experience depression, but you can reduce risk and strengthen resilience. A loving, stable environment, healthy routines, and early support make a big difference. And if depression does occur, remember—it is treatable, and children can recover with the right care.**

**Q11 What can I expect if my child has depression?**

**Response:**

**Learning that your child may have depression can feel overwhelming. The good news is that depression is treatable, and with the right support, children can recover and thrive. Knowing what to expect can help you feel more prepared and less alone.**

**🧩 What You May Notice in Your Child**

- **Emotional changes: Persistent sadness, irritability, or frequent tearfulness.**
- **Behavioral changes: Withdrawal from friends or activities, loss of interest in play or hobbies.**
- **Physical changes: Trouble sleeping, changes in appetite, low energy, or frequent unexplained aches and pains.**
- **School challenges: Difficulty concentrating, declining grades, or loss of motivation.**

**🌱 What to Expect During Treatment**

- **Professional evaluation: A pediatrician or mental health professional will assess your child’s symptoms and overall health.**
- **Therapy: Counseling (such as cognitive behavioral therapy or play therapy) helps children express feelings and learn coping skills.**
- **Family involvement: You’ll likely be part of the process—learning strategies to support your child at home.**
- **Medication (sometimes): In certain cases, antidepressants may be recommended, always with close monitoring.**
- **Gradual progress: Improvement often happens step by step. Some days will be better than others, and that’s normal.**

**💡 What You Can Do as a Caregiver**

- **Offer consistent love, patience, and reassurance.**
- **Keep routines and structure to help your child feel secure.**
- **Encourage healthy habits—sleep, nutrition, physical activity, and creative outlets.**
- **Stay in close contact with your child’s care team and school.**
- **Take care of your own well‑being, too—your strength helps your child heal.**

**🚨 When to Seek Immediate Help**

**If your child talks about self‑harm or not wanting to live, treat it as an emergency. Stay with them and call emergency services or the 988 Suicide & Crisis Lifeline (U.S.) or your local crisis helpline.**

**👉 Takeaway for caregivers: Expect ups and downs, but also expect hope. With professional care, family support, and patience, children with depression can recover and go on to live healthy, fulfilling lives.**

**Q12 What are the advanced treatments currently available for pediatric depression?**

**Response:**

**Depression in children is treatable, and while many children improve with counseling, family support, and sometimes medication, there are also advanced treatment options available when symptoms are more severe or do not respond to standard care. These treatments are always guided by specialists and tailored to each child’s needs.**

**🧩 Advanced and Emerging Treatment Strategies**

- **Combination Therapy**
  - **Using cognitive behavioral therapy (CBT) or other forms of psychotherapy together with antidepressant medication (such as SSRIs, carefully monitored) has been shown to be more effective for some children than either treatment alone.**
- **Interpersonal Therapy for Adolescents (IPT‑A)**
  - **A structured therapy that focuses on improving relationships and communication, which can reduce depressive symptoms.**
- **Family‑Focused Therapy**
  - **Involves parents and caregivers directly, teaching the whole family strategies to support the child and improve communication.**
- **School‑based Interventions**
  - **Collaboration with teachers and school counselors to provide academic accommodations, peer support, and structured coping strategies.**
- **Medication Advances**
  - **Selective serotonin reuptake inhibitors (SSRIs) remain the most studied and commonly prescribed medications for pediatric depression.**
  - **Ongoing research is exploring newer medications and safer dosing strategies for children and adolescents.**
- **Brain‑based and Neuromodulation Treatments (for severe, treatment‑resistant cases)**
  - **Repetitive Transcranial Magnetic Stimulation (rTMS): A non‑invasive procedure that uses magnetic fields to stimulate brain regions involved in mood regulation.**
  - **Electroconvulsive Therapy (ECT): Rarely used in children, but may be considered in life‑threatening or treatment‑resistant depression under strict medical supervision.**
  - **Ketamine and Esketamine (nasal spray): Emerging treatments being studied in adolescents for rapid relief of severe depression, though not yet widely approved for pediatric use.**
- **Lifestyle and Integrative Approaches**
  - **Structured exercise programs, mindfulness, and sleep interventions are increasingly recognized as important parts of advanced care plans.**

**💡 Important to Remember**

- **Advanced treatments are not first‑line options—they are considered when standard therapies are not enough.**
- **All treatments should be closely monitored by pediatric mental health specialists.**
- **Research in pediatric depression is growing, and new therapies are being studied to improve safety and effectiveness.**

**👉 Takeaway for caregivers: Most children respond well to counseling and, if needed, medication. But if symptoms are severe or persistent, specialists may recommend advanced treatments such as combination therapy, neuromodulation, or emerging medications. With the right care plan, children can recover and thrive.**

**Q13 Will the pediatric depression continue when the child became adult?**

**Response:**

**Not every child who experiences depression will continue to have it as an adult. However, research shows that childhood and adolescent depression can increase the risk of depression and other mental health challenges later in life if it is not recognized and treated early.**

**🔍 What Studies Show**

- **Children with depression are more likely to experience recurrent depression, anxiety, or substance use disorders in adulthood.**
- **Early depression has been linked to poorer health, difficulties in school and work, and challenges in relationships later on.**
- **Brain development during childhood and adolescence can be affected by untreated depression, which may increase vulnerability to future episodes.**

**🌱 The Good News**

- **Early treatment works. With counseling, family support, and—when needed—medication, many children recover fully and go on to live healthy, fulfilling adult lives.**
- **Protective factors—such as strong family support, healthy routines, positive friendships, and coping skills—can reduce the risk of depression continuing into adulthood.**
- **Even if depression returns later, children who received help early often have better tools and resilience to manage it.**

**💡 Takeaway for Caregivers**

**Pediatric depression does not always continue into adulthood, but it can if left untreated. The most important step you can take is to seek help early, stay engaged in treatment, and provide ongoing emotional support. With the right care, children can recover and build resilience that protects them well into adulthood.**

**Q14 How would I know if my child's depression is gone?**

**Response:**

**Recovery from depression in children is usually gradual, not an overnight change. Instead of looking for a single “all clear” moment, caregivers can watch for steady improvements in mood, behavior, and daily functioning.**

**🧩 Signs of Improvement**

- **Brighter mood: Your child smiles, laughs, or shows more positive emotions more often.**
- **Interest returns: They begin to enjoy activities, play, or hobbies they had lost interest in.**
- **Better energy: Less fatigue, more willingness to participate in school, family, or social activities.**
- **Improved focus: Schoolwork, concentration, and motivation start to get better.**
- **Healthier routines: Sleep and appetite become more regular.**
- **Stronger connections: They spend more time with friends and family, rather than withdrawing.**

**💡 Important to Remember**

- **Ups and downs are normal. Even after symptoms improve, children may still have occasional sad or irritable days.**
- **Recovery is a process. Some children may have one episode of depression and recover fully, while others may experience symptoms again later.**
- **Professional guidance matters. Your child’s doctor or therapist will help track progress and decide when treatment can be reduced or stopped.**
- **Ongoing support helps. Healthy routines, open communication, and family involvement remain important even after symptoms improve.**

**🚨 Watch for Warning Signs**

**If your child’s sadness, irritability, or withdrawal returns or worsens, or if they talk about self‑harm, seek professional help right away.**

**👉 Takeaway for caregivers: You’ll know your child is getting better when you see a return of joy, energy, and connection in their daily life. Stay in touch with their care team, celebrate progress, and remember that recovery is possible—and with continued support, children can thrive.**

**Q15 Does my child's depression impact their education level or employability?**

**Response:**

**Depression in children can affect many areas of life, including school performance and, later on, work opportunities. The impact is not automatic or permanent—with the right support and treatment, children can recover and thrive academically and professionally.**

**🎓 Impact on Education**

- **Concentration and memory problems: Depression can make it harder for children to focus, remember information, and complete assignments.**
- **Motivation and energy: Low energy or loss of interest may lead to missed classes, incomplete homework, or lower grades.**
- **School attendance: Some children may avoid school due to fatigue, anxiety, or social withdrawal.**
- **Risk of dropout: Untreated depression increases the risk of falling behind or leaving school early.**

**💼 Impact on Future Employability**

- **Early challenges: Struggles in school can sometimes carry over into difficulties with higher education or job readiness.**
- **Workplace functioning: Adults who had untreated depression as children may face challenges with confidence, stress management, or maintaining consistent performance.**
- **Resilience with treatment: Children who receive timely care often build coping skills that help them succeed in both education and employment later in life.**

**🌱 The Good News**

- **Treatment works: Counseling, family support, and—when needed—medication can significantly improve school performance and long‑term outcomes.**
- **School support matters: Teachers, school counselors, and accommodations (like extra time on assignments or flexible schedules) can help children stay on track.**
- **Skills for life: Learning coping strategies early can actually strengthen resilience, problem‑solving, and adaptability—qualities that benefit education and employability.**

**💡 Takeaway for Caregivers**

**Depression can affect a child’s education and, if left untreated, may influence future work opportunities. But with early treatment, school support, and family involvement, most children recover and go on to achieve their goals in school and beyond.**

**Response from DeepSeek (Model Name: 3.1 v)**

**Q1 What is depression in children?**

**Response:**

**### Understanding Depression in Your Child**

**It's completely normal to feel concerned when you hear the word "depression" in relation to your child. First and foremost, please know that childhood depression is a real, treatable medical condition—it is not a sign of weakness, a phase they will just "snap out of," or something you caused as a parent. Understanding it is the first and most important step toward helping your child feel better.**

**#### What Is It, Really?**

**At its core, depression (also known as Major Depressive Disorder) in children is more than just occasional sadness or moodiness. It’s a persistent feeling of deep sadness, irritability, or emptiness that interferes with a child's ability to function in their daily life—at school, at home, and with friends.**

**Think of it this way: While typical sadness comes and goes, depression is like a heavy cloud that follows a child everywhere, making it hard to enjoy things they used to love, find energy for the day, or feel hopeful about the future.**

**#### How Does It Look Different in Children?**

**Depression can sometimes look different in children than in adults. While adults often express profound sadness, children are more likely to show **irritability, anger, or frequent tantrums**. This is a key difference that is sometimes missed.**

**Here are some common signs to look for:**

*** **Emotional Changes:** Persistent sadness, hopelessness, irritability, anger, frequent crying, extreme sensitivity to criticism, or feelings of worthlessness (e.g., saying "I'm stupid" or "No one likes me").**

*** **Behavioral Changes:** Loss of interest in friends, hobbies, or activities they once enjoyed. Withdrawing from family and social events. Increased clinginess or, conversely, wanting to be alone all the time. Vocal outbursts or complaining.**

*** **Physical Changes:** Changes in appetite (eating much more or much less) or sleep patterns (trouble sleeping or sleeping too much). Low energy, frequent complaints of stomachaches or headaches that don't have a clear medical cause. Sluggishness or restlessness.**

*** **Cognitive Changes:** Difficulty concentrating, which often leads to a drop in grades at school. Trouble making decisions. Thoughts of death or suicide (this requires immediate attention).**

**#### What Causes Depression in Children?**

**There is no single cause. It's usually a combination of factors, including:**

*** **Biology:** Brain chemistry, hormones, and genetics can play a role. A family history of depression increases the risk.**

*** **Environment:** Stressful life events like family conflict, divorce, bullying, academic pressure, or the loss of a loved one can trigger depression.**

*** **Health:** Physical illnesses or chronic pain can contribute.**

**#### The Most Important Message: There Is Hope and Help**

**If you recognize these signs in your child, the most compassionate thing you can do is seek help from a professional.**

**1. **Start with Your Pediatrician:** They can rule out any underlying physical health issues and provide an initial assessment or referral.**

**2. **See a Mental Health Professional:** A child psychologist or psychiatrist specializes in diagnosing and treating childhood depression. They use proven methods like **therapy (talk therapy)**—which helps children understand and manage their feelings—and sometimes, if necessary, **medication**.**

**Your role as a caregiver is vital. You can support your child by:**

*** **Listening with love:** Let them know their feelings are valid and you are there for them without judgment.**

*** **Staying patient:** Recovery takes time. There will be good days and challenging days.**

*** **Encouraging healthy habits:** Gentle encouragement for regular sleep, healthy eating, and physical activity can help.**

**You are not alone in this. By asking this question, you've already taken a brave step toward helping your child. With your support and professional guidance, children with depression can and do get better.**

**Q2 What are the types of depression and mood disorders in children?**

**Response:**

**### Understanding Different Types of Depression and Mood Disorders in Children**

**It's wise to ask about the different types, as childhood depression isn't a one-size-fits-all condition. Understanding the specific patterns can help you and a professional better understand what your child is experiencing. Here are the most common types of depression and mood disorders seen in children.**

**#### 1. Major Depressive Disorder (MDD)**

**This is what people often think of as "clinical depression."**

*** **What it is:** It involves intense, persistent feelings of sadness, irritability, or emptiness that represent a clear change from a child's previous functioning.**

*** **Key Feature:** The symptoms (like low mood, loss of interest, changes in sleep/appetite) occur most of the day, nearly every day, for **at least two weeks**. These episodes can be severe and significantly disrupt school, home, and social life.**

**#### 2. Persistent Depressive Disorder (PDD) / Dysthymia**

*** **What it is:** This is a chronic, low-grade depressive mood that lasts for a much longer time.**

*** **Key Feature:** The symptoms are less severe than MDD but persist for **at least one year** in children. A child with PDD might seem consistently gloomy, irritable, or "down in the dumps." Because it's long-lasting, it can sometimes be mistaken for just part of the child's personality.**

**#### 3. Disruptive Mood Dysregulation Disorder (DMDD)**

*** **What it is:** This is a childhood-specific disorder characterized by severe and frequent temper outbursts that are grossly out of proportion to the situation.**

*** **Key Feature:** The core issue is **chronic, severe irritability**. Between outbursts, the child is persistently irritable or angry most of the day, nearly every day. DMDD was created to more accurately diagnose children who were previously (and often incorrectly) labeled with pediatric bipolar disorder.**

**#### 4. Adjustment Disorder with Depressed Mood**

*** **What it is:** This occurs when a child has a difficult time coping with a specific, identifiable stressful life event (e.g., a move, parents' divorce, changing schools, a loss).**

*** **Key Feature:** The depressive symptoms are a direct reaction to the stressor. They typically begin within three months of the event and usually ease within six months after the stressor ends. The distress is more significant than would normally be expected.**

**#### 5. Premenstrual Dysphoric Disorder (PMDD)**

*** **What it is:** For adolescent girls, PMDD involves severe mood symptoms (like extreme irritability, sadness, or anxiety) in the week or two before their period starts.**

*** **Key Feature:** Symptoms are cyclical and tied to the menstrual cycle, and they are much more severe than typical premenstrual syndrome (PMS).**

**#### Mood Disorders Involving "High" Moods (Mania/Hypomania)**

**These are more complex and require careful diagnosis by a specialist.**

**#### 1. Bipolar Disorder**

*** **What it is:** This involves distinct periods of depression (low moods) and periods of unusually elevated, expansive, or irritable moods called **mania** or **hypomania** (a less severe form).**

*** **Key Feature: Cycling between extremes.** During a "high" period, a child might show a sudden decrease in need for sleep, racing thoughts, talk very fast, be excessively risky, and have an inflated sense of self. **It is crucial to note that in children, manic episodes often present as intense irritability rather than euphoria.****

**### How This Information Helps You**

*** **It Highlights Patterns:** You might notice that your child's struggles fit a pattern—like constant low-grade grumpiness (suggesting PDD) or severe, explosive tantrums (suggesting DMDD).**

*** **It Guides Treatment:** The specific diagnosis matters because the most effective treatment can vary. For example, therapy is a cornerstone for all, but the approach might differ, and medication choices can be specific to the disorder (especially with bipolar disorder).**

*** **It Reduces Blame:** Understanding that these are defined medical conditions, not behavioral failures, can help reduce feelings of guilt or blame.**

**### The Most Important Step: A Professional Evaluation**

****Please use this information as a guide, not a tool for self-diagnosis.** These disorders have overlapping symptoms, and only a qualified mental health professional (like a child psychologist or psychiatrist) can conduct a thorough evaluation to determine the correct diagnosis.**

**Your role is to be a compassionate observer. Note the specific behaviors, moods, and how long they last. Share these observations with a professional. Getting the right diagnosis is the first step toward getting the right help, and with that, there is tremendous hope for your child's well-being.**

**Q3 What are risk factors for childhood depression?**

**Response:**

**### Understanding the Risk Factors for Childhood Depression**

**This is a very important question. Understanding the risk factors can help you see the whole picture of what might make a child more vulnerable to depression. It's crucial to remember that **having risk factors does not mean a child will definitely develop depression.** It simply means the odds are higher. Think of it like a scale: the more risk factors present, the heavier the scale tips toward vulnerability. The good news is that positive, supportive factors can help balance that scale.**

**Risk factors are generally grouped into a few categories:**

**#### 1. Biological and Genetic Factors**

*** **Family History:** A child with a parent or sibling who has depression or another mood disorder is at a higher risk. This is related to genetics and the brain chemistry that can be inherited.**

*** **Temperament:** Children who are naturally more withdrawn, have low self-esteem, or are prone to anxiety and worry may be more vulnerable.**

*** **Other Health Conditions:** Having a chronic illness (like diabetes or asthma), a learning disability, or ADHD can increase stress and isolation, contributing to depression.**

**#### 2. Environmental and Psychological Stressors**

**This is often the category where caregivers can most clearly see the pressures a child is facing.**

*** **Family Conflict:** Exposure to frequent arguing, tension, or a high-stress home environment is a significant risk factor.**

*** **Major Life Changes:** Events like parents' divorce, moving to a new school/area, or the loss of a loved one (including a pet) can be very difficult for a child to process.**

*** **Bullying or Social Rejection:** Persistent bullying, difficulty making friends, or feeling like an outsider at school can severely damage a child's sense of self-worth.**

*** **Academic Pressure:** Intense pressure to perform well academically can be a major source of chronic stress.**

*** **Trauma and Neglect:** Experiencing abuse (physical, emotional, or sexual), neglect, or other traumatic events is one of the strongest risk factors.**

**#### 3. Social and Peer-Related Factors**

*** **Social Isolation:** Having few or no friends to confide in can lead to feelings of loneliness and sadness.**

*** **Difficulties with Social Skills:** Children who struggle to read social cues or interact with peers may experience repeated negative social experiences.**

**### What's the Most Important Thing to Understand About Risk Factors?**

****Risk factors are not a checklist of blame.** They are pieces of a puzzle. A child can have several risk factors and not develop depression if they have strong protective factors. Conversely, a child with few apparent risk factors can still become depressed.**

**### The Power of Protective Factors**

**Just as there are risk factors, there are also things that can protect a child and build their resilience:**

*** **A Strong, Supportive Relationship with a Caregiver:** This is one of the most powerful protective factors. Simply knowing they have a safe, loving, and non-judgmental person to turn to makes a world of difference.**

*** **Positive Social Connections:** Having even one or two good friends and a sense of belonging at school or in an activity (like sports, art, or clubs).**

*** **Problem-Solving Skills:** Helping your child learn healthy ways to cope with setbacks and solve problems.**

*** **A Sense of Competence and Self-Esteem:** Encouraging your child's strengths and interests helps them feel capable and valued.**

*** **Access to Support Services:** Knowing that it's okay to seek help from school counselors, therapists, or pediatricians.**

**### What Should You Do With This Information?**

**Use it as a lens for understanding, not for worrying. If you see that your child has several risk factors and you are starting to notice signs of depression (like persistent sadness, irritability, or social withdrawal), it’s a signal to take action.**

**The best step is to talk to a professional, like your child’s pediatrician or a child therapist. They can help you understand your child's specific situation and provide guidance and support. You are not alone in this; understanding these risks is the first step toward providing the help and support your child needs.**

**Q4 What causes depression in children?**

**Response:**

**### Understanding the Causes of Depression in Children**

**This is one of the most heart-wrenching questions a parent or caregiver can ask. It's completely natural to search for a reason when you see your child in pain. Please hold onto this most important point first: **Childhood depression is a real medical condition, and it is never caused by one single thing, and it is never your fault.****

**Think of it like a scale. On one side, you have factors that make a child more vulnerable. On the other, you have strengths and supports that build resilience. Depression often develops when the vulnerable side becomes heavier than the resilient side.**

**Researchers believe several factors work together to cause depression. Here’s a breakdown of the main categories:**

**#### 1. Biology and Genetics (The "Wiring")**

*** **Brain Chemistry:** Our brains use chemicals called neurotransmitters to regulate mood, sleep, and appetite. When these chemicals are out of balance, it can lead to symptoms of depression.**

*** **Family History:** Depression can run in families. If a close family member (like a parent or sibling) has depression, a child may have a higher genetic risk. This isn't a guarantee they will get it, but it means they might be more vulnerable.**

*** **Hormones:** Changes in hormone levels can influence mood, which is part of why the risk of depression often increases during puberty.**

**#### 2. Temperament and Psychology (The "Thinking Style")**

*** **Inherent Temperament:** Some children are naturally more prone to anxiety, worry, or negative feelings. They may be more sensitive to criticism or setbacks.**

*** **Learned Patterns:** Children can develop a habit of negative thinking—believing that bad events are their fault, will last forever, and will ruin everything. This pattern can make them more susceptible to depression.**

**#### 3. Environment and Life Events (The "Triggers")**

**These are often the stressors we can see.**

*** **Family Stress:** High-conflict homes, divorce, or financial stress can create a heavy emotional burden for a child.**

*** **Trauma and Adversity:** Experiencing bullying, abuse, neglect, or the death of a loved one are significant triggers.**

*** **Social and Academic Pressure:** Struggling to make friends, feeling lonely, or facing intense pressure to succeed at school can be overwhelming.**

*** **Health Issues:** Dealing with a chronic illness like asthma or diabetes can contribute to feelings of sadness and isolation.**

**### The Most Important Takeaway: It's an Interaction**

**It's usually the *combination* of these factors that leads to depression. For example, a child with a genetic vulnerability (biology) might manage just fine until they face a major stressor like changing schools (environment). That combination can then tip the scales.**

**#### What This Means for You**

**1. **Let Go of Guilt:** It is crucial to understand that you did not cause your child's depression. The causes are complex and intertwined. Blaming yourself will only drain the energy you need to support your child.**

**2. **Focus on "What Now?" Instead of "Why?"** While understanding causes is helpful, the most powerful step is to focus on getting help. A professional's job isn't to assign blame but to understand these factors in your child's life and create a treatment plan to address them.**

**3. **You Are a Powerful Protective Factor:** Your love, support, and willingness to seek help are the strongest forces for your child's recovery. By getting support, you are adding weight to the resilient side of the scale.**

**The goal of understanding these causes is not to find a single villain but to build compassion and find the most effective path to help your child heal. With professional support and your care, children can and do recover from depression.**

**Q5 How do I know if my child is depressed?**

**Response:**

**It's understandable to feel worried when you notice changes in your child. Asking this question shows how much you care. It's important to know that childhood depression is more than just occasional sadness or moodiness; it's a persistent change that affects their daily life.**

**Here are some signs to look for, keeping in mind that every child is different. You are looking for a **cluster of these behaviors** that last for **at least two weeks** and represent a real change from your child's usual self.**

**#### Key Signs and Symptoms**

****1. Emotional Changes:****

*** **Persistent Sadness or Irritability:** This is a key sign. While adults often feel deep sadness, children with depression frequently appear consistently irritable, angry, or frustrated. They may have outbursts over seemingly small things.**

*** **Hopelessness:** They may make statements like, "What's the point?" or "Nothing ever gets better."**

*** **Worthlessness or Guilt:** They might be overly self-critical, saying things like, "I'm stupid," "No one likes me," or blaming themselves for things that aren't their fault.**

*** **Loss of Interest:** A major red flag is when they lose interest in hobbies, friends, or activities they once loved. They may drop out of sports teams or stop wanting to play with friends.**

****2. Behavioral Changes:****

*** **Social Withdrawal:** Pulling away from family and friends, spending much more time alone in their room.**

*** **Changes in Sleep Patterns:** This could be **insomnia** (trouble falling or staying asleep) or **hypersomnia** (sleeping too much, having a hard time getting up for school).**

*** **Changes in Appetite or Weight:** A noticeable increase or decrease in eating that leads to significant weight gain or loss.**

*** **Low Energy or Restlessness:** They may seem constantly tired, sluggish, or complain of having no energy. Conversely, they may seem agitated and unable to sit still.**

*** **Frequent Physical Complaints:** Unexplained headaches, stomachaches, or other pains that don't have a clear medical cause.**

*** **Difficulty Concentrating:** A drop in grades or teachers reporting that they can't focus in class.**

****3. Thoughts and Statements (That Require Immediate Attention)****

*** **Talking about Death or Suicide:** Any mention of wanting to die, not wanting to wake up, or being better off dead must be taken with the utmost seriousness. This is a medical emergency.**

**### What to Do if You Recognize These Signs**

**1. **Talk to Your Child Gently and Openly.****

*** Choose a calm, private moment.**

*** Use "I" statements. Instead of "You've been so moody," try, **"I've noticed you seem really sad/irritated lately, and I'm concerned about you. I love you, and I'm here to listen if you want to talk."****

*** Be patient. They might not open up right away. Let them know the door is always open.**

**2. **Schedule a Check-up with Your Pediatrician.****

*** This is a great first step. The doctor can rule out any physical health issues (like thyroid problems) that can mimic depression symptoms.**

*** They can also do a preliminary screening for depression and provide a referral to a mental health specialist.**

**3. **Seek a Evaluation from a Mental Health Professional.****

*** A child psychologist or psychiatrist is trained to diagnose childhood depression. They will talk with you and your child to get a full picture. Getting a proper diagnosis is the key to getting the right help.**

**### A Final, Important Note**

**Trust your instincts. You know your child better than anyone. If something feels wrong, it's better to have it checked out. **You are not overreacting.** Seeking help is a sign of strength and love, not failure. Early intervention can make a tremendous difference in your child's well-being.**

**Q6 How can I help my child with depression?**

**Response:**

**### How You Can Help Your Child with Depression: A Guide for Parents and Caregivers**

**First, please take a deep breath. The fact that you are asking this question means you are a loving, supportive caregiver, and that is one of the most powerful factors in your child's recovery. Helping a child with depression can feel overwhelming, but your role is not to "fix" them overnight. It's to be their steady, compassionate anchor as they navigate this difficult time.**

**Your support can be broken down into several key areas:**

**#### 1. Offer Unconditional Love and Emotional Support**

**This is the foundation of everything else.**

*** **Listen, Don't Just Solve:** Often, what your child needs most is to feel heard without judgment. Let them talk without immediately offering solutions or dismissing their feelings. You can say, "That sounds really hard. I'm here with you," or simply, "Tell me more about that."**

*** **Validate Their Feelings:** Avoid phrases like "Cheer up!" or "It's not that bad." Instead, acknowledge their pain. Say, "I can see how much this hurts," or "It makes sense that you feel that way." This tells them their feelings are valid and that they are not alone.**

*** **Be Patient:** Recovery from depression is a process with good days and bad days. There may be times when your child pushes you away. Try not to take it personally. Let them know you are there, consistently and patiently, no matter what.**

**#### 2. Take Practical Steps to Get Professional Help**

**Your love is essential, but depression is a medical condition that often requires professional treatment.**

*** **Schedule an Evaluation:** Start with your pediatrician to rule out any physical causes, and then seek a qualified child therapist (psychologist) or psychiatrist. Your role is to find the right professional and make sure your child gets to appointments.**

*** **Be a Partner in Treatment:** With your child's permission, communicate with their therapist. You can provide valuable insights about their behavior at home. Ask the therapist how you can best support the therapeutic work they are doing.**

*** **Support the Treatment Plan:** This may include ensuring your child takes any prescribed medication consistently and helping them practice the coping skills they learn in therapy.**

**#### 3. Create a Supportive and Low-Stress Environment at Home**

*** **Establish Routines:** Depression can make the world feel chaotic. Simple, predictable routines for meals, homework, and bedtime can provide a sense of safety and stability.**

*** **Encourage, Don't Force:** Gently encourage participation in positive activities, but don't force it. Instead of saying, "You have to go to the party," try, "Let's just drive by and see how you feel. We can leave anytime."**

*** **Focus on Small Wins:** Celebrate tiny accomplishments. Getting out of bed, taking a shower, or eating a full meal can be major victories. Acknowledge them with a simple, "I'm proud of you for doing that today."**

**#### 4. Prioritize Your Own Well-Being**

**This is not selfish; it's necessary.**

*** **You Can't Pour from an Empty Cup:** Supporting a child with depression is emotionally draining. If you are exhausted and overwhelmed, you will have less to give.**

*** **Find Your Own Support:** Talk to a trusted friend, join a support group for parents of children with mental health challenges, or consider therapy for yourself.**

*** **Practice Self-Care:** Make time for activities that recharge you, even if it's just for 15 minutes a day.**

**### What to Avoid**

*** **Minimizing their pain:** Avoid saying, "You're just being dramatic," or "It's a phase."**

*** **Blaming yourself or your child:** Depression is an illness, not a choice or a failure of parenting.**

*** **Comparing them to others:** "Your sister doesn't have this problem," is incredibly harmful.**

**### A Message of Hope**

**Your consistent, loving presence is a powerful force for healing. You are not alone in this journey. With professional help and your support, children can and do recover from depression. You are your child's greatest advocate.**

**Q7 Does my child need counseling for depression symptoms?**

**Response:**

**### Does My Child Need Counseling? A Guide for Making the Decision**

**This is a question that many caring parents and caregivers wrestle with. It's normal to feel unsure. The short answer is: **If you are asking this question, it is a strong sign that seeking a professional opinion is the right next step.****

**Think of it this way: If your child had a persistent fever that wasn't going away, you would likely take them to the doctor to find out what's wrong and how to treat it. Counseling is the same kind of supportive, professional help for emotional and mental health.**

**Here are some guidelines to help you decide if your child would benefit from counseling.**

**#### When Counseling Is Highly Recommended**

**Consider seeking a consultation with a child therapist if you notice several of the following signs, especially if they have lasted for **more than two weeks** and are interfering with your child's life:**

*** **The symptoms are persistent and intense.** The sadness, irritability, or anger isn't just a bad day; it's most of the day, nearly every day.**

*** **They are withdrawing from life.** They've lost interest in friends, hobbies, and activities they used to love.**

*** **There are significant changes in daily functioning.****

*** A noticeable drop in grades.**

*** Major changes in sleep (can't sleep or sleeps all the time) or appetite.**

*** Difficulty concentrating.**

*** **Your efforts to help aren't enough.** You've tried talking, spending more time together, and being supportive, but the problems seem too big to handle on your own.**

*** **Your child expresses hopelessness or worthlessness.** They make comments like, "Nothing matters," "I'm a bad person," or "No one would care if I was gone."**

*** **Your own instincts are telling you something is wrong.** You know your child better than anyone. If you feel in your gut that something is off, trust that feeling.**

**### Addressing Common Concerns**

*** **"Is this normal childhood moodiness?"** Normal moodiness comes and goes. Depression is a persistent state that doesn't lift easily and affects your child's ability to function.**

*** **"Will counseling make my child feel labeled or broken?"** A good child therapist specializes in making kids feel safe, understood, and normal. Counseling provides a toolbox of skills to manage big feelings—it’s about healing and strength, not weakness.**

*** **"Can't I just handle this as a parent?"** Your support is absolutely essential, but it's not a substitute for professional care. Just as you would see a dentist for a cavity, a therapist has specialized training to treat depression. Seeking help is a sign of great strength and love.**

**### What to Do Next**

**1. **Start with a Medical Check-up.** Schedule an appointment with your child's pediatrician. They can rule out any physical health issues (like thyroid problems or vitamin deficiencies) that can mimic depression symptoms. They can also provide an initial screening and a referral to a therapist.**

**2. **Schedule a Consultation with a Child Therapist.** Look for a licensed child psychologist or clinical social worker (LCSW) who specializes in childhood depression. The first appointment is often just for you (the parent) to talk about your concerns. This is a low-pressure way to get a professional's opinion.**

**3. **Talk to Your Child.** You can frame it in a positive and non-alarming way. For example:**

*** "I've noticed you've been having a really hard time lately, and I love you too much to watch you struggle. I found someone who is really good at helping kids work through tough feelings. Let's just go talk to them and see if it might help."**

*** "Think of it like a coach for your feelings. They can teach us some cool ways to feel better."**

**### The Bottom Line**

**You don't have to be 100% certain your child has depression to seek help. **The purpose of an evaluation is to answer the very question you're asking.** If the therapist determines your child is just going through a typical rough patch, you'll have peace of mind. If they diagnose depression, you've taken the crucial first step toward getting your child the effective support they need.**

**Q8 What treatment strategies would be useful for my children with depression?**

**Response:**

**### Treatment Strategies for Childhood Depression: A Guide for Parents and Caregivers**

**It's a sign of strength to ask about treatment options for your child. The good news is that childhood depression is very treatable. Effective treatment usually involves a combination of approaches, tailored specifically to your child's needs. Think of it as a toolkit—different strategies work together to help your child heal.**

**Here are the most common and effective treatment strategies used by professionals.**

**#### 1. Psychotherapy (Talk Therapy)**

**This is often the first and most important step. Therapy provides a safe space for your child to understand and express their feelings and learn coping skills. The most effective types for children are:**

*** **Cognitive Behavioral Therapy (CBT):** This is a widely used and highly effective approach. It helps children identify the connection between their thoughts, feelings, and behaviors. A CBT therapist will help your child:**

*** Challenge negative thought patterns (e.g., "I'm a failure").**

*** Develop more balanced and realistic ways of thinking.**

*** Learn problem-solving and coping skills to manage difficult emotions.**

*** **Interpersonal Therapy (IPT):** This therapy focuses on improving your child's relationships and social skills. It helps them address problems like conflicts with peers or family, social isolation, and grief, which can contribute to depression.**

*** **Play Therapy:** For younger children, play is their natural language. A play therapist uses toys, art, and games to help children express their feelings and work through problems they may not be able to verbalize.**

**#### 2. Medication**

**For moderate to severe depression, a doctor may recommend medication alongside therapy.**

*** **What it is:** Antidepressants, specifically **SSRIs (Selective Serotonin Reuptake Inhibitors)**, are the most commonly prescribed type for children. They work by helping to balance chemicals in the brain that influence mood.**

*** **Important Considerations:****

*** **Prescribed by a Specialist:** Medication should be prescribed and monitored by a child psychiatrist or a pediatrician experienced in childhood mental health.**

*** **Not a "Quick Fix":** It can take several weeks to see the full benefits.**

*** **Close Monitoring is Essential:** Your child's doctor will need to see them regularly, especially at the beginning, to check for any side effects and ensure the dosage is correct. The FDA recommends close monitoring for increased suicidal thoughts or behaviors when starting antidepressants in young people.**

*** **It's a Tool, Not a Replacement:** Medication is most effective when combined with therapy. It can help lift the cloud of depression enough for your child to fully engage in and benefit from therapy.**

**#### 3. Family Involvement and Support**

**You are a crucial part of the treatment team.**

*** **Parent Training or Family Therapy:** This involves you in the process. A therapist can teach you strategies to:**

*** Communicate more effectively with your child.**

*** Respond to depressive behaviors in a supportive way.**

*** Create a low-stress, structured, and nurturing home environment.**

*** **Your Role:** Your consistent support, patience, and encouragement are invaluable. Your job is to love them, ensure they attend appointments, and help them practice the skills they learn in therapy.**

**#### 4. Lifestyle and Environmental Strategies**

**These are supportive measures that can make a significant difference in your child's overall well-being.**

*** **Routine:** Establishing predictable daily routines for meals, homework, and bedtime provides a sense of security.**

*** **Healthy Habits:** Gently encourage regular physical activity (even a short walk), a balanced diet, and adequate sleep. These all have a direct impact on mood.**

*** **Social Connection:** Help your child maintain social connections, but without pressure. Facilitate low-stakes interactions, like having one friend over for a short time.**

*** **School Collaboration:** Talk to your child's school counselor or teacher. They can provide support and accommodations (like a reduced workload during difficult periods) to help reduce academic stress.**

**### The Treatment Process: What to Expect**

**1. **Comprehensive Evaluation:** A mental health professional will conduct a thorough assessment to make an accurate diagnosis.**

**2. **Creating a Plan:** You, your child (depending on their age), and the therapist will work together to create a treatment plan with clear goals.**

**3. **Patience and Consistency:** Recovery is a process, not an event. There will be ups and downs. Consistency with therapy and other treatments is key.**

**4. **Ongoing Communication:** Maintain open communication with your child's treatment team.**

**### A Final Message of Hope**

**Finding the right treatment strategy may take some time, but it is a journey worth taking. With the right combination of professional support, family love, and patience, your child can regain their joy and build resilience for the future.**

**Q9 How is depression diagnosed in children?**

**Response:**

**### How Depression is Diagnosed in Children: A Guide for Parents and Caregivers**

**It's completely understandable to wonder how a professional can determine if a child has depression. The process isn't like taking a blood test; instead, it's a comprehensive evaluation that gathers information from several sources to see the full picture. The goal is to understand your child's unique experience and rule out other possible causes for their symptoms.**

**Here is a step-by-step breakdown of what you can expect during the diagnostic process:**

**#### Step 1: The Professional Evaluation (The "Who")**

**The diagnosis should be made by a qualified mental health professional who specializes in children, such as a:**

*** **Child Psychologist****

*** **Child Psychiatrist****

*** **Pediatric Clinical Social Worker (LCSW) or Licensed Professional Counselor (LPC)****

**Often, the first step is a visit to your **pediatrician** to rule out any medical issues.**

**#### Step 2: The Components of the Assessment (The "How")**

**The professional will gather information through a few key methods:**

****1. Clinical Interview with You (the Parents/Caregivers):****

**This is a crucial part. The professional will ask you detailed questions about:**

*** **Your child's symptoms:** What you've observed (sadness, irritability, sleep changes, etc.).**

*** **Timeline:** When the symptoms started, how long they've lasted, and how they've impacted daily life.**

*** **Family history:** Whether there is a history of depression or other mood disorders in the family.**

*** **Home and school life:** Any major stressors, changes, or challenges.**

****2. Clinical Interview with Your Child:****

**The therapist will talk with your child in an age-appropriate way. They might use games, drawings, or simple conversations to help your child express their feelings. They will ask about their mood, friendships, school, and home life.**

****3. Standardized Questionnaires and Rating Scales:****

**Both you and your child (if they are old enough) may be asked to fill out brief questionnaires. These tools help quantify symptoms (e.g., rating sadness on a scale of 1 to 5) and ensure the professional is assessing all the necessary criteria consistently.**

****4. Gathering Information from School:****

**With your permission, the professional may contact your child's teacher or school counselor. They can provide valuable insight into how your child is functioning in a different social and academic setting.**

**#### Step 3: What the Professional is Looking For (The "Criteria")**

**The professional will compare all the gathered information to the standard diagnostic criteria for **Major Depressive Disorder** (or other related conditions like Persistent Depressive Disorder). For a diagnosis, a child must have experienced **five or more of the following symptoms** for **at least two weeks**, and this must represent a change from their previous functioning. At least one of the symptoms must be either (1) depressed mood or (2) loss of interest or pleasure.**

*** Depressed or irritable mood most of the day, nearly every day.**

*** Markedly diminished interest or pleasure in all, or almost all, activities.**

*** Significant weight loss or gain, or decrease or increase in appetite.**

*** Insomnia (difficulty sleeping) or hypersomnia (sleeping too much).**

*** Fatigue or loss of energy.**

*** Feelings of worthlessness or excessive guilt.**

*** Difficulty thinking, concentrating, or making decisions.**

*** Recurrent thoughts of death or suicide.**

**#### Step 4: Ruling Out Other Causes**

**A critical part of the diagnosis is ensuring the symptoms aren't better explained by something else. The professional will work to rule out:**

*** **Medical conditions** (e.g., thyroid problems, vitamin deficiencies).**

*** **Other mental health conditions** (e.g., anxiety disorders, ADHD, trauma-related disorders).**

*** **Normal grief** after a significant loss.**

**### What You Can Do to Prepare**

*** **Take Notes:** Before the appointment, write down the specific behaviors and changes you've noticed, including when they started.**

*** **Be Open and Honest:** Share your concerns openly, even if they are difficult to talk about. The more information the professional has, the more accurate the assessment will be.**

*** **Talk to Your Child:** Explain that you're going to see a "feelings doctor" or a "worries expert" who helps kids feel better. Reassure them that they are not in trouble and that you are on this journey together.**

**### The Outcome: A Path Forward**

**The purpose of a diagnosis is not to just assign a label. It's to create a clear understanding of what your child is experiencing so that you can develop an **effective treatment plan**. A diagnosis opens the door to the right kind of help, whether it's therapy, lifestyle changes, or other supports.**

**Remember, you are an essential partner in this process. Your observations and insights are invaluable in helping your child get the support they need.**

**Q10 Can I prevent my child from developing depression?**

**Response:**

**### Can I Prevent My Child from Developing Depression?**

**This is a question that comes from a place of deep love and care. It shows how much you want to protect your child's well-being. The honest answer is that while there is no guaranteed way to *prevent* depression entirely—especially since factors like genetics and brain chemistry play a role—you can significantly **reduce your child's risk and build their resilience.****

**Think of it not as building a wall to keep depression out, but as strengthening your child's foundation so they are better able to weather life's inevitable storms.**

**Here are the most powerful, proactive steps you can take to build your child's mental health resilience:**

**#### 1. Foster a Strong, Nurturing Connection**

**You are your child's primary source of security. This connection is their greatest buffer against stress.**

*** **Unconditional Love and Acceptance:** Ensure your child knows your love does not depend on their achievements or behavior. They need to feel valued for who they are.**

*** **Open Communication:** Create an environment where all feelings are welcome. Let them know it's safe to feel sad, angry, or scared, and that you are there to listen without immediately trying to "fix" it. Use phrases like, "It sounds like you're feeling really disappointed. Tell me more about that."**

**#### 2. Actively Teach Coping and Emotional Skills**

**Resilience is the ability to bounce back from setbacks. You can help them develop this muscle.**

*** **Name and Validate Emotions:** Help your child identify what they're feeling. "It looks like you're feeling frustrated because that puzzle is tricky." This helps them understand their inner world.**

*** **Model Healthy Coping:** Let your child see you managing your own stress in healthy ways—taking a walk, talking about your feelings, or taking a deep breath. You are their most important model.**

*** **Problem-Solve Together:** When challenges arise, guide them through the process of finding solutions instead of solving it for them. This teaches them they can handle difficulties.**

**#### 3. Create a Supportive and Predictable Environment**

*** **Establish Routines:** Consistent routines for meals, homework, and bedtime provide a sense of safety and predictability, which lowers anxiety.**

*** **Encourage Healthy Habits:** Prioritize regular physical activity, a balanced diet, and adequate sleep. Physical health and mental health are deeply connected.**

*** **Monitor and Manage Stress:** Be aware of the pressures in your child's life (academic, social, extracurricular) and help them find a healthy balance. It's okay to scale back if it's too much.**

**#### 4. Be Proactive During Times of Change or Stress**

**Life events like moving, divorce, or starting a new school can be risky periods.**

*** **Provide Extra Support:** Check in more frequently and be patient.**

*** **Consider Short-Term Support:** Even a few sessions with a child therapist during a tough transition can provide them with valuable tools and a safe space to talk.**

**#### 5. Know the Signs and Seek Help Early**

**This is one of the most crucial forms of prevention.**

*** **Trust Your Instincts:** You know your child best. If you notice persistent changes in their mood, behavior, or functioning, don't wait.**

*** **Early Intervention is Key:** Seeking guidance from a pediatrician or therapist at the first signs of struggle is like addressing a small crack before it becomes a larger one. It is a sign of strength, not failure.**

**### A Final, Important Note**

**Please release yourself from the pressure of total prevention. You cannot control everything. Some children, despite having the most loving and supportive families, may still develop depression due to biological factors.**

****Your goal is not to be a perfect parent who prevents all pain. Your goal is to be the connected, attuned parent who raises a resilient child.** By focusing on building a strong relationship and teaching coping skills, you are giving your child the best possible tools for a lifetime of mental wellness.**

**Q11 What can I expect if my child has depression?**

**Response:**

**### What to Expect When Your Child Has Depression: A Guide for the Journey**

**First, please know that it's normal to feel a mix of fear, sadness, and uncertainty. Asking this question is a brave first step. Understanding what may lie ahead can help you feel more prepared and less alone. While every child's experience is unique, there are some common patterns you can expect.**

**This journey is often not a straight line, but with support, there is a clear path forward.**

**#### 1. The Emotional and Behavioral Landscape**

**You will likely see a continuation of the symptoms that led to the diagnosis, but now with a new context. Expect:**

*** **Good Days and Bad Days:** Recovery is not linear. Your child may have a few good days followed by a very difficult one. This is normal and doesn't mean the treatment isn't working. It's important to celebrate the small wins without being discouraged by the setbacks.**

*** **Irritability as Much as Sadness:** Remember that in children, depression often looks like anger, frustration, and irritability. They may snap at you or have meltdowns over seemingly small things. Try not to take it personally; it's the illness talking.**

*** **Withdrawal and Isolation:** Your child may continue to pull away from family and friends. They are not rejecting you; they are often overwhelmed, exhausted, or feel like a burden.**

*** **Apathy and Lack of Motivation:** Don't be surprised if they show little interest in things they used to love. This "anhedonia" (inability to feel pleasure) is a core symptom of depression, not a personal choice.**

**#### 2. The Treatment Process**

*** **It Takes Time:** Therapy and/or medication do not work overnight. It can take several weeks to see noticeable improvements. Patience is critical.**

*** **Therapy Requires Work:** Counseling is not a passive process. Your child will be learning new skills, and it will be important to gently encourage them to practice these skills at home. There might be times they resist going to sessions.**

*** **You Are Part of the Team:** The therapist will likely want to involve you periodically. You may be asked to help reinforce strategies at home or provide updates on your child's progress. Your insights are invaluable.**

**#### 3. Your Role as a Caregiver: The Balancing Act**

*** **Your Emotions Matter Too:** Watching your child struggle is heartbreaking. You may feel guilt, anger, helplessness, and exhaustion. **These feelings are normal.** It is essential to take care of your own mental health through your own support system, therapy, or respite care. You cannot pour from an empty cup.**

*** **Shift from "Fixing" to "Supporting":** Your role is not to "cure" your child's depression but to be their stable, loving anchor. Your consistent presence is more powerful than any immediate solution you can offer.**

*** **Adjust Your Expectations:** You may need to temporarily adjust expectations around schoolwork, chores, and social commitments. The goal is to reduce pressure while still maintaining a basic structure.**

**#### 4. The Long-Term Outlook: There Is Hope**

*** **Depression is Treatable:** With the right support and treatment, the vast majority of children with depression see significant improvement. They can return to enjoying their lives, friendships, and activities.**

*** **They Learn Invaluable Skills:** Through therapy, your child isn't just treating depression; they are learning lifelong skills for managing stress, understanding their emotions, and building resilience that will serve them well into adulthood.**

*** **Your Relationship Can Deepen:** Navigating this challenge together, with honesty and compassion, can ultimately strengthen the bond between you and your child.**

**### A Final, Reassuring Message**

**This path requires patience, compassion, and a great deal of strength—from both you and your child. There will be challenging moments, but there will also be moments of hope and progress. Trust the process, trust your child's treatment team, and most importantly, trust the love you have for your child. That love is the steady foundation upon which their recovery will be built.**

**Q12 What are the advanced treatments currently available for pediatric depression?**

**Response:**

**### Advanced Treatments for Pediatric Depression: A Guide for Families**

**When a child is struggling with depression and initial treatments haven't brought the relief they need, it's natural to look for other options. It's important to know that the field of child psychiatry is continually advancing, and there are indeed specialized treatments available for complex or treatment-resistant cases.**

****A crucial starting point:** These "advanced" treatments are typically considered when a child has not responded adequately to **evidence-based first-line treatments**, such as cognitive-behavioral therapy (CBT) or other forms of psychotherapy, and/or trials of antidepressant medication. They are conducted by highly specialized teams, often in academic or tertiary care centers.**

**Here are some of the advanced treatment strategies currently available:**

**#### 1. Neuromodulation Therapies (Brain Stimulation Therapies)**

**These treatments directly target brain circuits involved in mood regulation. They are used very carefully in children and adolescents, typically under strict protocols.**

*** **Transcranial Magnetic Stimulation (TMS):****

*** **What it is:** A non-invasive procedure that uses magnetic fields to stimulate nerve cells in the prefrontal cortex, an area of the brain linked to mood regulation. The patient is awake and alert during the sessions, which last about 20-40 minutes.**

*** **For Pediatrics:** TMS has received FDA clearance for treating major depressive disorder in adolescents (typically ages 12-18) who have not responded to at least one antidepressant medication. It is generally well-tolerated with minimal side effects (the most common being mild scalp discomfort).**

*** **Electroconvulsive Therapy (ECT):****

*** **What it is:** Often misunderstood, modern ECT is a safe and highly effective medical procedure performed under general anesthesia. It involves a brief, controlled electrical stimulation of the brain.**

*** **For Pediatrics:** ECT is used **very rarely** in youth and only for the most severe, life-threatening, or treatment-resistant cases of depression (e.g., when a child is catatonic, psychotic, or at extreme immediate risk of suicide). A team of specialists must determine that the potential benefits significantly outweigh the risks.**

**#### 2. Intensive Treatment Programs**

**These programs provide a higher level of care than weekly therapy.**

*** **Intensive Outpatient Programs (IOP) and Partial Hospitalization Programs (PHP):****

*** **What they are:** These programs allow a child to receive structured, multi-modal treatment for several hours a day, multiple days a week, while returning home each evening. They combine multiple forms of therapy (individual, group, family) and skills training in a supportive environment.**

*** **Goal:** To stabilize symptoms and prevent the need for inpatient hospitalization.**

*** **Residential Treatment Centers:****

*** **What they are:** For children with the most severe and chronic symptoms, a residential setting provides 24-hour therapeutic care in a structured environment. This allows for constant support and intensive work on deep-seated issues.**

**#### 3. Novel and Emerging Pharmacological Approaches**

*** **Ketamine and Esketamine (Spravato®):****

*** **What it is:** Ketamine is a medication that works on a different brain system (the glutamate system) than traditional antidepressants. A nasal spray form, esketamine, is FDA-approved for **treatment-resistant depression in adults**.**

*** **For Pediatrics:** The use of ketamine and esketamine in children is still considered **investigational**. It is sometimes used in very specialized, often hospital-based, settings for severe, treatment-resistant cases, but it is not a standard treatment. Research is ongoing to determine its safety and efficacy in adolescents.**

**#### 4. Complementary and Integrative Approaches**

**These are often used *alongside* traditional treatments to support overall well-being.**

*** **Comprehensive Lifestyle Management:** This goes beyond simple advice. Specialized programs may focus on designing specific exercise regimens, nutritional plans, and sleep hygiene protocols that are tailored to a child's neurobiology to support mood regulation.**

*** **Mindfulness-Based Therapies:** Advanced applications of mindfulness and meditation are increasingly integrated into treatment to help children manage difficult emotions and thoughts.**

**### The Most Important Step: A Specialized Assessment**

**If you are considering advanced treatments, the essential next step is a **comprehensive evaluation by a child and adolescent psychiatrist at a specialized academic or tertiary care center.** They can:**

**1. Confirm the diagnosis and ensure all other factors have been considered.**

**2. Determine if your child has truly exhausted standard, first-line treatments.**

**3. Discuss whether an advanced treatment is appropriate, considering the potential benefits, risks, and the specific needs of your child.**

****A Message of Hope****

**The existence of these advanced options means there is always hope, even in complex situations. The goal of these treatments is to provide a breakthrough, to reduce symptoms enough that your child can re-engage effectively in therapy and life. You are your child's best advocate, and seeking out this information is a powerful act of love.**

**Q13 Will the pediatric depression continue when the child became adult?**

**Response:**

**That's a very important and understandable question. Many parents and caregivers wonder about the long-term journey for their child.**

**The short answer is: **It's not a guarantee, but childhood depression can increase the risk for depression in adulthood.** However, it's crucial to know that many children with depression recover and do not experience it as adults.**

**Think of it this way: successfully treating your child's depression now is one of the most powerful things you can do to support their long-term mental health. Early intervention can change the course of their life.**

**Here’s a more detailed breakdown:**

**### Why Depression Might Continue**

*** **Underlying Vulnerability:** Some individuals have a biological or genetic predisposition to depression. This vulnerability can be a lifelong factor.**

*** **Chronic Stressors:** If the underlying causes of the depression (like ongoing family stress, bullying, or trauma) are not resolved, the symptoms may persist.**

*** **Severity:** Children with more severe, recurrent, or long-lasting episodes of depression may have a higher likelihood of experiencing it later in life.**

**### Reasons for Hope and Recovery**

*** **Effective Treatment:** When a child receives proper treatment—such as therapy (like CBT), and sometimes medication—they learn crucial coping skills. These skills don't just help them now; they become tools they can use for the rest of their lives to manage stress and negative emotions.**

*** **Brain Plasticity:** A child's brain is still developing and is very "plastic," meaning it can adapt and change. Effective intervention can help shape healthier neural pathways for managing emotions.**

*** **Support Systems:** A strong, supportive, and understanding family environment is a protective factor that significantly improves the chances of long-term well-being.**

**### What You Can Do Now to Make a Difference**

**Your role is incredibly important. Focus on these key actions:**

**1. **Prioritize Consistent Treatment:** The most critical step is ensuring your child stays engaged with their current treatment plan. This includes attending therapy sessions regularly and following any medication plans as prescribed by their doctor.**

**2. **Keep Communication Open:** Create a safe space where your child feels comfortable talking about their feelings without judgment. Let them know it's okay to not be okay, and that you are there for them.**

**3. **Learn the Warning Signs:** Be aware of the signs of recurring depression (e.g., social withdrawal, changes in sleep or appetite, irritability, talk of hopelessness). Catching a new episode early leads to quicker intervention.**

**4. **Focus on Overall Wellness:** Encourage healthy habits that support mental health for everyone: good nutrition, regular physical activity, adequate sleep, and time for fun and relaxation.**

**5. **Take Care of Yourself:** Caring for a child with depression can be challenging. Make sure you also have support, whether it's from a partner, friend, therapist, or support group for caregivers. You can't pour from an empty cup.**

****In summary, a history of pediatric depression is a risk factor, but it is not a life sentence.** With love, support, and appropriate treatment, your child can build resilience and have a bright, healthy future.**

**Q14 How would I know if my child's depression is gone?**

**Response:**

**That is a wonderful and very important question. It shows how attentive and hopeful you are for your child's recovery. It's important to know that recovery from depression is often a process, not a single "on/off" switch. We look for a return to a child's normal, healthy baseline.**

**Instead of thinking of it as "gone," it can be more helpful to think of it as **"in remission" or "managed."** This means the symptoms have significantly faded and your child has the tools to handle life's ups and downs.**

**Here are some key signs that your child is doing much better and their depression is in remission:**

**### 1. A Return to Their Normal Self**

**This is the most significant sign. You'll see the child you know coming back.**

*** **Enjoying Life Again:** They re-engage with hobbies, activities, and friendships they once enjoyed. They might laugh genuinely and seek out fun.**

*** **Energy and Motivation:** They have the energy for daily tasks, schoolwork, and socializing. The heavy feeling of "I can't" is replaced with "I can" or even "I want to."**

*** **Stable Mood:** The intense sadness, irritability, or emptiness has lifted. Their mood is more stable, and they can experience a full range of emotions appropriate to situations (happiness, disappointment, excitement) instead of being stuck in a negative state.**

**### 2. Positive Behavioral Changes**

*** **Healthy Habits:** Their sleep and appetite return to a normal pattern for them. They're not sleeping too much or too little, and they're eating regularly.**

*** **Improved Concentration:** They can focus on schoolwork, conversations, and tasks without being overwhelmed by distraction or fatigue.**

*** **Social Re-engagement:** They start reaching out to friends, making plans, and showing interest in social activities instead of isolating themselves.**

**### 3. Using Coping Skills**

**This is a great long-term indicator. You might notice your child using the skills they learned in therapy *on their own* when they face a setback or feel stressed. For example, they might take a deep breath when frustrated, go for a walk to clear their head, or challenge a negative thought.**

**---**

**### Important Things to Keep in Mind:**

*** **"Good Days" vs. Sustained Recovery:** Everyone has bad days, including kids. A bad day is normal. Recovery is when the bad days are the exception, not the rule, and your child can bounce back from them.**

*** **Talk with the Therapist:** The professional working with your child is your best partner in assessing this. They will evaluate symptoms against a clinical scale and can give you a more objective opinion on your child's progress. **This is a conversation you should have directly with them.****

*** **The Goal is Management, Not Perfection:** The ultimate goal isn't to never feel sad again. It's to equip your child to manage difficult emotions effectively so that a bad day doesn't spiral into a depressive episode.**

**### A Word of Caution: Be Aware of the "Smile"**

**Sometimes, a child may appear better on the surface—they might be "smiling" and functioning—but internally, they are still struggling. This is why open communication is vital. Keep checking in gently, not just by asking "Are you happy?" but with questions like:**

*** "How have you been feeling lately?"**

*** "What's been on your mind?"**

*** "I've noticed you seem more like yourself, and I'm so glad. How does it feel to you?"**

****Your next step:** The best way to know for sure is to **schedule a conversation with your child's therapist.** You can share your observations and ask them: "Based on your sessions, do you feel my child's depression is in remission?" Together, you can create a plan for ongoing support and what to do if symptoms return.**

**Q15 Does my child's depression impact their education level or employability?**

**Response:**

**That's a very understandable and important question. The short answer is: **yes, untreated or poorly managed depression can impact a child's education and, by extension, future employability.** However, and this is the most crucial part, **this outcome is not inevitable.** With proper support and treatment, these impacts can be minimized, and your child can absolutely achieve their academic and career goals.**

**Let's break down the "how" and, more importantly, the "what we can do about it."**

**### How Depression Can Impact Education**

**Depression is more than just sadness; it affects the brain's ability to function. In school, this can look like:**

*** **Difficulty Concentrating:** Your child may struggle to focus in class, follow instructions, or complete assignments. Their mind might feel "foggy."**

*** **Memory Problems:** Retaining new information for tests or recalling lessons can become much harder.**

*** **Loss of Motivation & Energy:** The fatigue and apathy that come with depression make it incredibly difficult to find the drive to do homework, study, or even attend school.**

*** **Absenteeism:** Due to fatigue, physical symptoms (like headaches or stomachaches), or anxiety, your child may miss a significant amount of school.**

*** **Social Withdrawal:** They may stop participating in class discussions or group projects, missing out on collaborative learning.**

*** **Irritability:** This can lead to conflicts with teachers and peers, creating a negative school environment.**

**Over time, these challenges can lead to falling grades, being held back, or a general disconnection from learning. This can affect their confidence and limit their options for higher education or specific career paths.**

**### The Connection to Future Employability**

**The skills developed in school—like showing up consistently, meeting deadlines, managing tasks, and working with others—are the same skills needed in the workplace. If depression interferes with developing these skills, it can create challenges later on.**

**However, the goal of treatment is to build **resilience** and **coping strategies**. These are incredibly valuable skills in *any* career.**

**### The Most Important Message: What You Can Do to Mitigate These Impacts**

**This is where the power of intervention comes in. You are not powerless. Here’s how you can help:**

**1. **Prioritize Treatment:** The single most effective thing you can do is ensure your child is consistently engaged in their treatment plan (therapy, medication if prescribed). Effective treatment directly addresses the symptoms that interfere with learning.**

**2. **Communicate with the School:****

*** **Request a Meeting:** Schedule a meeting with your child's teacher(s), school counselor, and/or principal.**

*** **Develop a 504 Plan or IEP:** You can work with the school to create a **504 Plan** or an **Individualized Education Program (IEP)**. These are formal plans that provide accommodations to level the playing field. Examples include:**

*** Extended time on tests and assignments.**

*** Breaks during the day when feeling overwhelmed.**

*** A reduced homework load when necessary.**

*** Permission to leave the classroom to see the counselor.**

*** Preferential seating.**

**3. **Focus on Strengths and Interests:** Help your child maintain connections to activities they enjoy and are good at, even if it's not academic. This builds self-esteem and reminds them that they are more than their depression.**

**4. **Teach Self-Advocacy:** As they get older, help them learn to articulate their needs respectfully. Being able to say, "I'm struggling with my focus today, may I have a short break?" is a vital skill for school and future employment.**

****In summary, while depression presents real challenges to learning, it does not define your child's potential.** Many highly successful people have managed depression throughout their lives. The strategies they learn now to cope with their illness—such as perseverance, self-awareness, and asking for help—can become their greatest strengths.**

****Your next step:** Discuss these concerns with your child's therapist, who can provide specific guidance. Then, contact your child's school counselor to start a conversation about what support systems are available.**

**Response from ChatGPT (GPT-5)**

**Q1 What is depression in children?**

**Response:**

**Depression in children is more than just feeling sad or having a bad day. All kids experience ups and downs, but depression is when those feelings of sadness, hopelessness, or irritability last for weeks or longer and start to affect the child’s daily life.**

**A child with depression might:**

- **Seem unusually sad, tearful, or irritable most of the time**
- **Lose interest in activities they used to enjoy**
- **Struggle with schoolwork or concentration**
- **Have changes in sleep or appetite**
- **Show low energy or fatigue**
- **Withdraw from friends or family**
- **Say things that show feelings of worthlessness or hopelessness**

**It’s important to know that depression is not a sign of weakness or poor parenting. It’s a real health condition that can happen in children, just like in adults. The good news is that with proper support, treatment, and understanding, children with depression can feel better and thrive.**

**If you notice these signs in your child and they last for more than two weeks, it may be time to reach out to a healthcare provider for support and guidance.**

**Q2 What are the types of depression and mood disorders in children?**

**Response:**

**Children and teens can experience several kinds of mood disorders. These conditions affect the way they feel, think, and act. The most common types include:**

- **Major depressive disorder (MDD): Ongoing feelings of sadness, hopelessness, or loss of interest in things they once enjoyed. Symptoms last most of the day, nearly every day, for at least two weeks.**
- **Persistent depressive disorder (dysthymia): A milder, but longer-lasting form of depression. Children may seem irritable or down most of the time for a year or more.**
- **Bipolar disorder: Shifts between periods of depression and episodes of unusually high energy, mood, or irritability (called mania or hypomania).**
- **Disruptive mood dysregulation disorder (DMDD): Frequent, intense temper outbursts that are not typical for the child’s age, along with a consistently irritable or angry mood between outbursts.**
- **Adjustment disorder with depressed mood: Sadness or hopelessness that develops after a stressful life event, such as moving, divorce, or loss, but usually improves once the child adjusts or receives support.**

**Each child’s experience may look different, and symptoms can overlap. What’s most important is noticing lasting changes in mood, energy, sleep, school performance, or relationships and seeking help when needed.**

**With the right treatment and support, children with mood disorders can feel better and build healthy coping skills.**

**Q3 What are risk factors for childhood depression?**

**Response:**

**Depression doesn’t have a single cause. Instead, it often develops from a mix of biological, emotional, and environmental factors. Some things that may increase a child’s risk include:**

- **Family history: If a parent or close relative has depression or another mental health condition, the child may be more likely to experience it.**
- **Stressful life events: Experiences like bullying, divorce, moving, loss of a loved one, or family conflict can trigger or worsen depression.**
- **Chronic illness or pain: Ongoing health problems may increase feelings of sadness, frustration, or isolation.**
- **Trauma or abuse: Experiencing neglect, abuse, or other trauma can deeply affect a child’s emotional well-being.**
- **Low self-esteem or negative thinking patterns: Children who struggle with self-worth or who tend to be very self-critical may be more vulnerable.**
- **School or social challenges: Academic struggles, learning difficulties, or feeling left out by peers can contribute.**

**It’s important to remember: having risk factors does not mean a child will definitely develop depression. Many children with risk factors stay emotionally healthy. But knowing these risks helps families and providers watch more closely and get help early if needed.**

**Q4 What causes depression in children?**

**Response:**

**Depression in children doesn’t come from one single cause. Instead, it usually develops from a combination of factors that affect how a child feels, thinks, and responds to stress. Some possible causes include:**

- **Biological factors: Differences in brain chemicals (neurotransmitters) or brain development may make some children more likely to develop depression.**
- **Genetics: Depression can run in families. If a parent or close relative has depression or another mental health condition, a child may be at higher risk.**
- **Stressful or traumatic events: Loss of a loved one, divorce, moving, bullying, or abuse can trigger depression.**
- **Medical conditions: Chronic illness, pain, or certain medications may play a role.**
- **Personality and coping style: Children who are very self-critical, have low self-esteem, or find it hard to manage stress may be more vulnerable.**
- **Environment: Ongoing conflict at home, school struggles, or lack of support can increase the risk.**

**It’s important to know that depression is never the child’s fault—or the parent’s fault. It is a medical condition, not a weakness, and with the right care and support, children with depression can feel better.**

**Q5 How do I know if my child is depressed?**

**Response:**

**It’s normal for children to feel sad, frustrated, or moody at times. But depression is different—it lasts longer (two weeks or more), happens most of the day nearly every day, and begins to interfere with your child’s daily life.**

**You may notice signs such as:**

- **Mood changes: Your child seems sad, tearful, hopeless, or unusually irritable most of the time.**
- **Loss of interest: They no longer enjoy activities, hobbies, or time with friends.**
- **School difficulties: Trouble concentrating, a drop in grades, or refusing to go to school.**
- **Changes in daily habits: Sleeping too much or too little, eating much more or less than usual, or low energy.**
- **Withdrawal: Avoiding friends, family, or social activities.**
- **Negative thoughts: Talking about worthlessness, guilt, or feeling like life isn’t worth living.**

**If you notice several of these changes lasting more than two weeks, it may be more than just a phase. Trust your instincts. If something feels off, it’s worth reaching out to your child’s doctor or a mental health professional.**

**The most important thing to remember is that depression is treatable, and early support can make a big difference.**

**Q6 How can I help my child with depression?**

**Response:**

**Supporting a child with depression can feel overwhelming, but you play an important role in their healing. Here are some ways you can help:**

- **Listen and validate their feelings: Let your child know it’s okay to talk about their emotions. Avoid dismissing their sadness or saying they should “just snap out of it.”**
- **Seek professional help: Reach out to your child’s doctor or a mental health provider. Therapy, counseling, and sometimes medication can make a big difference.**
- **Stay involved and connected: Spend time together doing activities they enjoy, even small ones. Consistent support helps them feel less alone.**
- **Encourage healthy habits: Regular sleep, physical activity, and balanced meals support both mood and overall health.**
- **Work with the school: Teachers and counselors can provide support and help with any academic struggles.**
- **Model coping skills: Show healthy ways of managing stress, like deep breathing, talking about feelings, or taking breaks.**
- **Watch for warning signs: If your child talks about self-harm or suicide, take it seriously and get immediate help (call emergency services or a crisis line).**

**Remember: depression is not your fault or your child’s fault. With treatment, patience, and love, children can and do get better.**

**Q7 Does my child need counseling for depression symptoms?**

**Response:**

**If your child is showing signs of depression—such as ongoing sadness, irritability, loss of interest in activities, changes in sleep or appetite, or withdrawal from friends and family—counseling can be very helpful.**

**Counseling (sometimes called therapy) gives children a safe space to talk about their feelings and learn healthy coping skills. A trained mental health professional can also help parents understand what their child is going through and offer guidance on how to support them at home.**

**You should especially consider counseling if:**

- **The symptoms last two weeks or more**
- **Your child’s mood or behavior is interfering with school, friendships, or family life**
- **Your child expresses feelings of hopelessness, worthlessness, or talks about self-harm**

**Getting help early can prevent symptoms from getting worse and helps your child feel better sooner. Even if you’re unsure whether your child has depression, counseling can still provide valuable support.**

**If you’re worried about your child’s safety (such as talk of suicide or self-harm), seek help immediately—call emergency services or a crisis hotline.**

**Remember: reaching out for counseling is a sign of strength and care, not weakness. With the right support, children with depression can heal and thrive.**

**Q8 What treatment strategies would be useful for my children with depression?**

**Response:**

**The good news is that depression in children is treatable. The right plan often combines professional care with strong support at home and school. Common strategies include:**

- **Counseling/Therapy:**
  - ***Cognitive Behavioral Therapy (CBT)* helps children identify and change negative thought patterns.**
  - ***Interpersonal Therapy (IPT)* focuses on improving relationships and coping with life changes or stress.**
  - **Play therapy or family therapy may also be recommended, depending on your child’s age and needs.**
- **Medication (if needed):
  In some cases, a doctor may prescribe antidepressant medication—usually along with therapy. Medication can help balance brain chemicals that affect mood. A specialist will carefully monitor your child for safety and effectiveness.**
- **Family support:**
  - **Listening without judgment and encouraging open conversations about feelings.**
  - **Maintaining routines, including regular sleep, meals, and physical activity.**
  - **Working with your child’s school to ensure they get the right support.**
- **Healthy lifestyle habits:**
  - **Exercise, outdoor activities, and time with supportive friends or family.**
  - **Balanced nutrition and good sleep routines.**
  - **Limiting screen time if it interferes with rest or mood.**
- **Crisis support:
  If your child talks about self-harm or suicide, take it seriously. Seek help right away—call emergency services or a crisis hotline.**

**Every child is different, so treatment should be tailored to your child’s age, symptoms, and needs. With the right care, children with depression can improve and develop healthy coping skills for the future.**

**Q9 How is depression diagnosed in children?**

**Response:**

**Depression in children is diagnosed by a healthcare professional—usually a pediatrician, child psychiatrist, or psychologist—after a careful evaluation. There is no single test, like a blood test or scan, that can confirm depression. Instead, the process includes:**

- **Detailed interview: The provider talks with the child and caregiver about symptoms, behaviors, feelings, and changes in daily life.**
- **Medical history and exam: This helps rule out other health conditions that might cause similar symptoms (like thyroid problems, sleep disorders, or certain medications).**
- **Screening tools or questionnaires: Age-appropriate checklists may be used to measure mood, behavior, and functioning at home or school.**
- **Input from others: Teachers or other caregivers may be asked about what they see in the classroom or social settings.**

**To make a diagnosis, symptoms usually need to:**

- **Last for at least two weeks**
- **Happen most of the day, nearly every day**
- **Interfere with school, friendships, or family life**

**It’s important to know: a diagnosis is not a label, but a first step toward getting the right support and treatment. With early identification and care, children with depression can feel better and thrive.**

**Q10 Can I prevent my child from developing depression?**

**Response:**

**It’s not always possible to prevent depression, because many factors—like genetics, brain chemistry, and stressful life events—are outside of anyone’s control. However, there are ways you can lower your child’s risk and strengthen their ability to cope with challenges:**

- **Build strong connections: Spend regular quality time together and encourage open conversations about feelings.**
- **Support self-esteem: Praise effort, not just results, and help your child notice their strengths.**
- **Encourage healthy habits: Regular sleep, balanced meals, and physical activity all support emotional health.**
- **Model coping skills: Show your child how you handle stress in healthy ways (deep breathing, problem-solving, asking for help).**
- **Maintain routines: Predictable daily routines provide children with a sense of security.**
- **Help them manage stress: Teach age-appropriate skills for handling disappointment, conflict, and pressure.**
- **Stay connected to school and peers: Encourage friendships, hobbies, and positive school involvement.**
- **Seek support early: If you notice ongoing changes in your child’s mood or behavior, don’t wait—reach out to a healthcare provider. Early support can often prevent symptoms from becoming more serious.**

**Remember: You cannot prevent every challenge your child may face—but you can give them the tools, support, and love they need to get through tough times. And if depression does develop, effective treatments and supports are available.**

**Q11 What can I expect if my child has depression?**

**Response:**

**If your child is diagnosed with depression, it’s natural to feel worried or uncertain. The good news is that depression is treatable, and with the right support, most children improve and go on to thrive. Here’s what you can expect:**

- **Treatment takes time: Progress may be gradual. Some children start to feel better within a few weeks of treatment, while for others it may take longer. Consistency and patience are important.**
- **Ups and downs are normal: Even with treatment, your child may still have hard days. What matters is the overall improvement over time.**
- **Family involvement is key: You play a vital role—by offering support, encouraging healthy routines, and participating in therapy when needed.**
- **School and social life may be affected: Your child’s mood and energy might make school or friendships harder at times. Working closely with teachers and counselors can help provide extra support.**
- **Treatment options may vary: Counseling (therapy), lifestyle changes, and sometimes medication are used together. Your child’s care plan will be personalized to their needs.**
- **Recovery is possible: Many children with depression recover fully, especially when treatment starts early. They also build coping skills that help them handle stress in the future.**

**Most importantly: having depression does not define your child. With care, support, and patience, your child can feel better and live a full, healthy life.**

**Q12 What are the advanced treatments currently available for pediatric depression?**

**Response:**

**Most children with depression respond well to standard treatments like counseling (therapy), healthy lifestyle changes, and sometimes medication. But when symptoms are more severe or don’t improve with these approaches, specialists may consider advanced treatment options. These are usually provided by child psychiatrists in specialized settings and may include:**

- **Medication management: If one antidepressant doesn’t help, a doctor may try a different type or adjust the dose. Sometimes a combination of medications is used, always with careful monitoring for safety.**
- **Intensive outpatient or partial hospitalization programs: These provide more structured support than weekly therapy, often including several hours of therapy per day while the child continues living at home.**
- **Inpatient care (hospitalization): In very severe cases—especially if there is risk of self-harm—short-term hospitalization may be needed to keep the child safe and start treatment in a secure environment.**
- **Emerging/advanced options (for treatment-resistant depression):**
  - ***Transcranial Magnetic Stimulation (TMS):* A non-invasive procedure that uses gentle magnetic pulses to stimulate areas of the brain linked to mood.**
  - ***Ketamine or esketamine therapy:* These medications may be considered in rare, severe cases, usually in research or highly specialized centers.**
  - ***Electroconvulsive Therapy (ECT):* Very rarely used in children, but may be considered in extreme cases where nothing else has worked.**

**These advanced treatments are only considered when standard approaches haven’t been effective, and decisions are always made carefully with parents and specialists working together.**

**The most important step is early recognition and treatment. Most children improve with therapy, family support, and (if needed) carefully monitored medication—advanced treatments are reserved for the small number of children who need extra help.**

**Q13 Will the pediatric depression continue when the child became adult?**

**Response:**

**Not every child who experiences depression will carry it into adulthood. Some children have a single episode of depression that improves with treatment and support, while others may experience recurring episodes as they grow.**

**What we know:**

- **Early treatment helps: Children who receive care—such as therapy, medication if needed, and family support—are less likely to have ongoing or severe depression later in life.**
- **Some risk remains: Having depression in childhood does increase the chance of depression returning in adolescence or adulthood, especially if risk factors like family history, trauma, or ongoing stress are present.**
- **Skills can last a lifetime: Children who learn coping strategies, stress management, and healthy routines through therapy often carry those tools into adulthood, which lowers the risk of relapse.**
- **Monitoring is important: Even after symptoms improve, regular check-ins with healthcare providers during the teen years and beyond can help catch and treat new episodes early.**

**The most important thing to remember is: pediatric depression does not mean your child will struggle forever. With treatment, support, and healthy coping skills, many children recover fully and go on to live happy, fulfilling adult lives.**

**Q14 How would I know if my child's depression is gone?**

**Response:**

**Recovery from depression looks different for every child, but there are some common signs that symptoms are improving or have resolved. You may notice that your child:**

- **Has a brighter mood: They seem less sad, irritable, or withdrawn most of the time.**
- **Shows interest again: They start enjoying activities, hobbies, or time with friends that they used to avoid.**
- **Improves in daily life: School performance, concentration, and participation in family routines get better.**
- **Has stable routines: Sleeping, eating, and energy levels return to healthier patterns.**
- **Talks more positively: They express more hope, confidence, or self-worth, and fewer negative or hopeless thoughts.**
- **Copes better with stress: When challenges come up, they use healthy coping strategies instead of shutting down or withdrawing.**

**It’s also common for recovery to happen gradually, with good days and harder days. That doesn’t necessarily mean the depression is “back”—ups and downs are a normal part of healing.**

**Your child’s healthcare provider can help assess whether the depression is in remission (symptoms have improved significantly) or recovery (symptoms are gone and functioning is back to normal). Regular follow-up visits are important to make sure progress is lasting.**

**Most importantly: celebrate the progress, no matter how small. Healing from depression takes time, but many children recover fully and build resilience for the future.**

**Q15 Does my child's depression impact their education level or employability?**

**Response:**

**Depression can affect many areas of a child’s life, including school and future opportunities—but it does not mean your child cannot succeed in education or employment. Here’s what to know:**

- **In the short term: Depression may make it harder for children to concentrate, keep up with assignments, or attend school regularly. They may also feel less motivated or confident. With the right support—like counseling, academic accommodations, and family encouragement—most children are able to catch up and succeed.**
- **In the long term: If left untreated, ongoing depression can affect learning, skill-building, and confidence, which may influence future education and job opportunities. However, with early treatment and consistent support, many children recover fully and go on to thrive in college, careers, and adult life.**
- **Employability: Depression itself does not prevent someone from working. In fact, many people with a history of childhood depression do well in meaningful jobs. Learning coping strategies, building resilience, and having strong support systems often make children stronger problem-solvers as adults.**

**The most important factor is treatment. Children who get help early—through therapy, healthy routines, school support, and sometimes medication—are much more likely to stay on track academically and build the skills they need for future employment.**

**Remember: your child’s diagnosis does not define their future. With care and encouragement, children with depression can learn, grow, and achieve their goals just like anyone else.**
